# Deep phenotyping of T cell populations under long‐term treatment of tacrolimus and rapamycin in patients receiving renal transplantations by mass cytometry

**DOI:** 10.1002/ctm2.629

**Published:** 2021-11-08

**Authors:** Yiyang Li, Huimin An, Chuan Shen, Boqian Wang, Ting Zhang, Yifan Hong, Hui Jiang, Peijun Zhou, Xianting Ding

**Affiliations:** ^1^ State Key Laboratory of Oncogenes and Related Genes School of Biomedical Engineering Institute for Personalized Medicine Shanghai Jiao Tong University Shanghai People's Republic of China; ^2^ Division of Kidney Transplant Department of Urology Ruijin Hospital Shanghai Jiao Tong University School of Medicine Shanghai People's Republic of China; ^3^ Department of Liver Surgery Renji Hospital Shanghai Jiao Tong University School of Medicine Shanghai People's Republic of China

**Keywords:** immune system, mass cytometry, rapamycin, renal transplantation, tacrolimus

## Abstract

Tacrolimus (FK506) and rapamycin (RAPA) are widely used to maintain long‐term immunosuppression after organ transplantation. However, the impact of accumulative drug administration on the recipients’ immune systems remains unclear. We investigated the impact of 3‐year FK506 or RAPA treatment after renal transplantation on the human immune systems. A discovery cohort of 30 patients was first recruited, and we discovered two distinctive T lineage suppressive regulatory patterns induced by chronic treatment of FK506 and RAPA. The increased percentage of senescent CD8^+^CD57^+^ T lineages and less responsive T cell receptor (TCR) pathway in the FK506 group indicate better graft acceptance. Meanwhile, percentages of regulatory T cells (Tregs) and expression of CTLA‐4 were both up to two‐fold higher in the RAPA group, suggesting the inconsistent reactivation potential of the FK506 and RAPA groups when an anti‐tumour or anti‐infection immune response is concerned. Additionally, up‐regulation of phosphorylated signaling proteins in T lineages after in vitro CD3/CD28 stimulation suggested more sensitive TCR‐signaling pathways reserved in the RAPA group. An independent validation cohort of 100 renal transplantation patients was further investigated for the hypothesis that long‐term RAPA administration mitigates the development of tumours and infections during long‐term intake of immunosuppressants. Our results indicate that RAPA administration indeed results in less clinical oncogenesis and infection. The deep phenotyping of T‐cell lineages, as educated by the long‐term treatment of different immunosuppressants, provides new evidence for personalized precision medicine after renal transplantations.

## Graphical abstract

Graphical Highlights

1. Different impacts of FK506 and RAPA on immune systems of renal transplantation recipients lead to different cell subsets involved in immunosuppression.

2. The suppressive subsets in the FK506 group are mainly CD57^+^ senescent T cells, percentage of which was about half in the RAPA group, and TCR signaling pathway is less likely to be activated under CD3/CD28 stimulation.

3. Meanwhile, the percentage of regulatory T cells and the expression of CTLA‐4 were more abundant in the RAPA group.

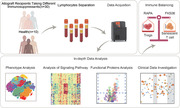

## 1 INTRODUCTION

Renal transplantation remains the most effective treatment for end‐stage renal diseases in terms of mortality, quality of life and health care savings.^1^ Immunosuppressants are necessary to prevent allograft rejection and suppress the activities of immune system in recipients.^2^, ^3^ Calcineurin inhibitors (CNIs) and mammalian targets of rapamycin (mTOR) inhibitors are widely used in clinical transplantation, resulting in the long‐term survival of allografts.^4^, ^5^, ^6^, ^7^, ^8^ CNIs inhibit T cell receptor (TCR)‐induced calcineurin/nuclear factor of activated T cells (NFAT) translocation to block interleukin (IL)‐2 transcription or to directly interfere with the NFAT/forkhead/winged‐helix transcription factor P3 (FOXP3) interaction.^9^ Tacrolimus (FK506), the most dominant immunosuppressant in clinical practice, is a representative CNI that leads to a long‐term increased risk of viral infection and induces a reduction in the proportion of CD4^+^ Tregs within the peripheral blood mononuclear cells (PBMCs).^10^, ^11^, ^12^ Rapamycin (RAPA) directly binds to mTORC1 and inhibits the PI3K/AKT/mTOR signaling pathway, which is part of the CD28 costimulatory and IL‐2 receptor signaling pathways generating full effector T‐cell activation.^5^, ^6^, ^7^, ^8^ RAPA is beneficial for the generation of Tregs, which may favour long‐term graft survival.^13^

The triple immunosuppressive protocol, FK506 or RAPA in combination with anti‐proliferative agents and corticosteroids, is widely used in transplant recipients. The immunosuppressant scheme used to be determined after taking safety, efficacy, intensity of rejection and graft acceptance into consideration.^2^ Along with the increased overall survival, allograft recipients receiving different long‐term (at least 3 years after transplant) maintenance immunosuppression therapies becomes common. Long‐term complications associated with the choice of different immunosuppressive regimens occurred. Therefore, the formulation of the immunosuppressive protocol should not only rely on the clinical outcome or immunopharmacological arguments but also the immune landscape shaped by different immunosuppressants.^14^ Previous studies mostly focused on identifying specific cell types by flow cytometry or changes in immune subsets within 1 year after renal transplantation. However, systemic investigation focusing on the immune phenotypes and functions shaped chronically by FK506 and RAPA is lacking.^15^

Herein, single‐cell mass cytometry (CyTOF)^16^, ^17^, ^18^, ^19^ was applied to comprehensively characterize the immune phenotype of immune cells in renal allograft recipients taking immunosuppressants. Our findings depict the deep phenotyping of T lineages and intracellular signaling networks induced by FK506 and RAPA and provide a fundamental understanding of the immune system alteration chronically induced by these regimens. Furthermore, our article provides rationale and perspective evidence‐based guidance for long‐term personalized medicine when the personalized immunosuppressive protocol was initially determined.

## 2 RESULTS

### 2.1 High‐dimensional CyTOF‐based profiling of health controls and renal graft recipients maintained on long‐term immunosuppressants

**FIGURE 1:**
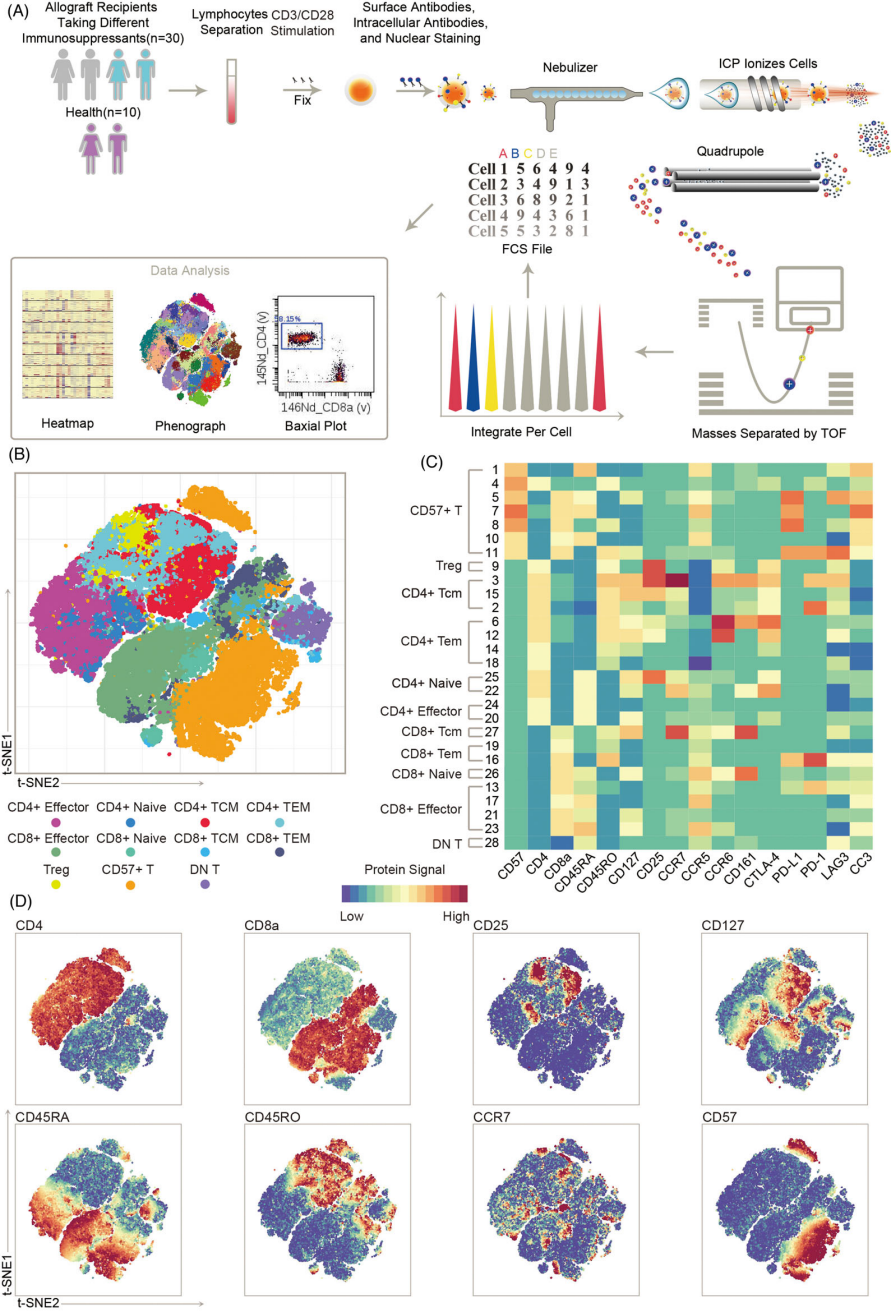
High‐dimensional profiling of health controls and renal graft recipients using CyTOF. (A) A schematic pipeline to present the procedures applied on profiling peripheral blood mononuclear cells (PBMCs) of healthy controls and renal graft recipients by CyTOF. PBMCs are extracted from peripheral blood, stimulated by CD3/CD28, stained with metal‐conjugated antibodies, and analyzed using CyTOF. CD45^+^CD3^+^ viable PBMCs were gated for downstream analysis, including clustering and signaling protein analysis by FlowSOM and PhenoGraph. (B) t‐distributed stochastic neighbor embedding (t‐SNE) visualization of merged CD45^+^CD3^+^ T cells (127 242 in total) derived from PBMCs (*n* = 40) with labelled cluster partitions. (C) Heatmap shows mean expression values of surface proteins, normalized per column by z‐score. (D) t‐SNE visualization of T cells illustrating the expression of eight proteins used for identifying subpopulations. Colour in (C) and (D) represent the ArcSinh‐transformed signal intensity of the indicated markers. TEM, effector memory T cells; TCM, central memory T cells; DN T, CD4^−^CD8^−^ T cells; Treg, regulatory T cell; CTLA‐4, cytotoxic T‐lymphocyte‐associated protein 4; PD‐L1, programmed death‐ligand 1; PD‐1, programmed cell death protein 1; CC3, cleaved caspase‐3; LAG3, lymphocyte activation gene‐3

A CyTOF panel of 29 metal isotope‐tagged monoclonal antibodies was designed to acquire a global overview of the T‐cell network in the immune system and simultaneous downstream intracellular signaling cascades under CD3/CD28 stimulation (Figure 1A). The panel contained 17 T‐cell lineage surface markers to distinguish the major subsets of T‐cell lineage and 12 intracellular signaling markers. PBMCs were processed within 1 h after being collected from patients who received renal transplantation to remain as close as possible to in vivo conditions. Table 1 listed the clinical characteristics of the samples. PBMCs received CD3/CD28 stimulation to activate TCR signaling pathways related to the immune system of patients with renal transplantation. Samples were collected before and 2 min after stimulation. With this panel and time points, we analyzed single‐cell PBMC suspensions of patients who took FK506 (*n* = 16) or RAPA (*n* = 14) for over 3 years and health controls (*n* = 10). This study was approved by the Ethics Committees of the Ruijin Hospital (IRB Number: *KY2018‐153*), as described in the *Method and Material* section (Figure S1). To discriminate among T‐cell lineage subsets, live and single CD45^+^ cells were discriminated with DNA staining and event length (Figure S2).

**TABLE 1:** Clinical information of the 30 patients in the discovery cohort

Characteristics of the sample			
Demographics^a^			
Gender (male/female)	FK506	6/10	
	RAPA	5/9	
Age (years)	FK506	46 (27‐63)	
	RAPA	52 (32‐74)	
Body mass index (kg/m^2^)	FK506	21.2 (15.6‐23.8)	
	RAPA	21.9 (16.9‐31.6)	
Survival time after surgery (years)	FK506	8 (3‐21)	
	RAPA	10 (7‐14)	
Immunosuppressor use^b^			
Immunosuppressor (mg/day)	FK506	MMF^#^	1000‐1500
		Steroid^#^	5‐7.5
		FK506	3.47 (1‐5)
	RAPA	MMF^#^	1000‐1500
		Steroid^#^	5‐7.5
		RAPA	0.78 (0.25‐2)

Abbreviations: MMF, mycophenolate mofetil; RAPA, rapamycin.

^a^ Values indicate the number of patients or average and range.

^b^ Milligram equivalent of oral tacrolimus or rapamycin respectively. The dose is the average cumulative dose per day.

^#^ Doses of MMF and steroids were not significantly different between the FK506 and RAPA groups

To profile the phenotype of immune cells, all CD45^+^CD3^+^ cells from each sample were downsampled to 1 × 10^4^, and t‐distributed stochastic neighbor embedding (tSNE) was used to reduce high dimensional data onto a two‐dimensional plane for visualization (Figure S3). According to the similarity of cell surface markers signal, the FlowSOM^20^ and PhenoGraph^21^ analysis were applied to identify 28 clusters (Figure 1B, Figures S4 and S5A). The 28 clusters identified by PhenoGraph were preliminarily categorized into 11 major phenotypic subpopulations by their surface marker expression profiles (Figure 1C and Figure S5B). These 11 major phenotypic subpopulations include CD57^+^ T cells, CD4^+^ Tregs (CD4^+^CD25^+^CD127^−^), and CD4^+^CD57^−^ or CD8^+^CD57^−^ effector (CD45RA^+^CCR7^−^), naïve T cells (CD45RA^+^ CCR7^+^), T_EM_ (CD45RO^+^ CCR7^−^) and T_CM_ (CD45RO^+^ CCR7^+^), which were preliminarily identified by CD57, CD4, CD8, CD45RA, CD45RO, CCR7, CD25 and CD127 (Figure 1D and Figure S5C).

### 2.2 Percentages of suppressive T‐cell subpopulations are enhanced in renal graft recipients with long‐term immunosuppressants treatment after renal transplantation

**FIGURE 2:**
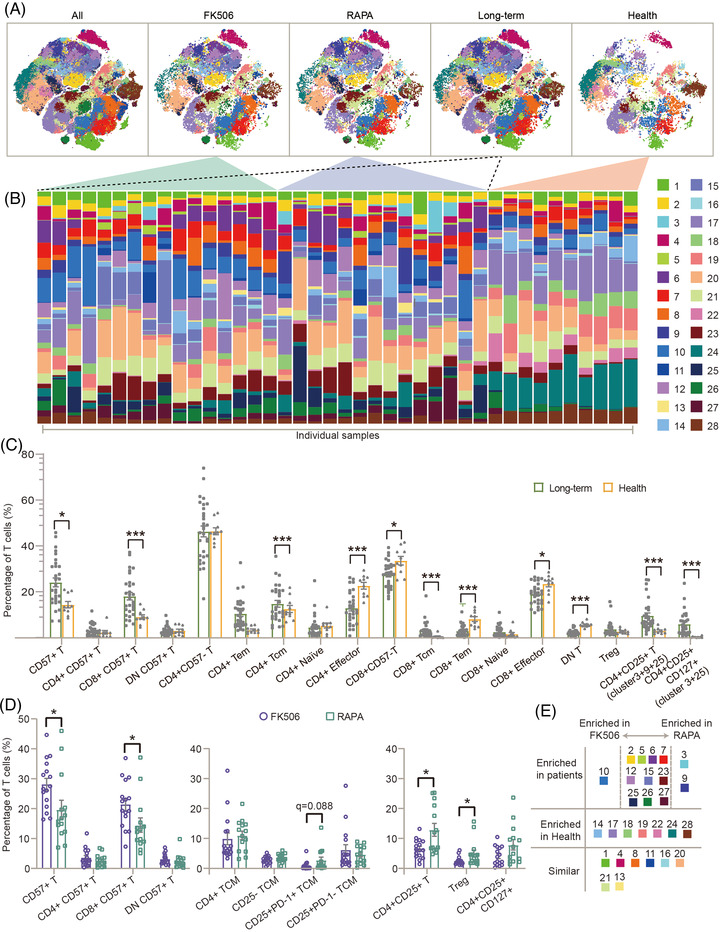
PhenoGraph meta‐clustering reveals an augment of suppressive subpopulations in renal graft recipients. (A) t‐distributed stochastic neighbor embedding (tSNE) maps of all samples, including the healthy controls (*n* = 10), long‐term groups (*n* = 30), FK506 (*n* = 16) and rapamycin (RAPA) (*n* = 14) groups, were coloured by cluster numbers. The long‐term groups consisted of the FK506 and RAPA groups. The bars at the top of the heatmap show the group panel. (B) The portions of the 28 clusters identified by PhenoGraph in T cells are demonstrated with bar plots for each sample. The colours of the 28 metaclusters in panel B correspond to those of the tSNE maps in panel (A). (C) Percentages of 18 phenotypic subpopulations, merged from the 28 clusters according to the Figure 1B‐D, represent phenotypically distinct immune cell subpopulations between long‐term and health controls. Error bars indicate mean ± s.e.m. *q < 0.05, ***q < 0.005. (D) Distinct subpopulations between FK506 and RAPA. Error bars indicate mean ± s.e.m. *q < 0.05. All *q*‐values were corrected *p*‐values by Benjamini–Hochberg adjustment and *p*‐values were calculated using unpaired Mann–Whitney test. (E) A table summarized the clusters that were enriched in either long‐term or health samples, or similar in both groups. Enrichment in the long‐term groups was further divided into FK506 and RAPA groups. Statistical analysis was performed using unpaired Mann–Whitney test and Benjamini–Hochberg adjustment. The colours of the 28 metaclusters in panel (E) correspond to those of the tSNE maps in panel (A)

The spatial representations of healthy controls and stable renal‐graft recipients (long‐term) maintained by two different immunosuppressant combinations, composed of mycophenolate‐mofetil, steroids and either FK506 or RAPA, are distinct (Figure 2A and Figure S6). Unstimulated samples (*n* = 40) were applied to phenotype analyses in Figure 2. The t‐SNE map of long‐term groups was formed by merging CD45^+^CD3^+^ cells in the FK506 and RAPA groups. Frequencies of T cell clusters of each individual also represent the differences between healthy controls and long‐term samples (Figure 2B). To investigate the difference and similarity between healthy controls and long‐term treatments, frequencies of 28 clusters and 18 phenotypical subpopulations were compared. The identified 18 phenotypical subpopulations incorporated the 11 major phenotypic subpopulations presented in Figure 1 and seven extra phenotypical subpopulations, including CD4^+^CD57^−^ T, CD8^+^CD57^−^ T, CD4^+^CD25^+^ T, CD4^+^CD25^+^CD127^+^ T (CD4^+^CD25^+^ non‐Tregs), CD4^+^CD57^+^ T, CD8^+^CD57^+^ T, DN CD57^+^ T subpopulations. The correspondence between the 28 clusters and the identified 18 phenotypical subpopulations is listed in Table S1. We observed that No.14, No.18, No. 19 clusters (T_EM_), No.17 and No.24 (effector), No.22 (CD4^+^ naïve) and No.28 (DN T) were enriched in healthy controls compared with the long‐term treatment groups (Figure S7). Meanwhile, frequencies of No.5, No.7 and No.10 clusters (CD8^+^CD57^+^), No.15 and No.27 clusters (T_CM_), No.3 and No.25 clusters (CD4^+^CD25^+^), No.6 and No.12 clusters (T_EM_) and No.26 cluster (naïve) and No.23 cluster (effector) were 0.1 to two‐fold higher in the long‐term groups compared with healthy controls (Figure S7). Of note, the most distinct clusters in the long‐term groups are CD8^+^CD57^+^, T_CM_ and CD4^+^CD25^+^ T‐cell clusters. We used the classical hand‐gating method to separate 18 phenotypical subpopulations, providing independent validation of the FlowSOM analysis (Figure 2C and Figure S8). Statistical analysis of manual‐gating subpopulations was consistent with the FlowSOM analysis. Previous literature exploring markers for T lineage noted that cells with CD57^+^ had reported senescent or suppressor capacities, associated with maintaining graft acceptance in the recipient.^22^, ^23^ Compared with healthy controls, CD4^+^CD25^+^ T lineages and T_CM_ showed higher abundances in the long‐term groups, caused by continuous antigen stimulation.^24^ Therefore, frequencies of CD57^+^ T cells, CD4^+^CD25^+^ T cells, T_CM_ and their subpopulation were compared respectively between FK506 and RAPA groups (Figure 2D). We observed immune cells of subsets, which were responsible for maintaining graft acceptance, were different in the FK506 and RAPA groups. CD57^+^ T subsets, senescent and terminally differentiated T cells, were about 15% higher in the FK506 group, especially the No.10 cluster (CD8^+^CD57^+^). Frequencies of CD4^+^CD25^+^ cells, including Tregs, were two‐fold higher in the RAPA group than in the FK506 group. Of note, senescent T cells and Tregs are beneficial for the establishment of graft acceptance after transplantation.^10^, ^22^ The frequencies of Tregs and CD4^+^CD25^+^CD127^+^ (CD4^+^CD25^+^ non‐Tregs) were compared among the healthy controls, FK506 and RAPA groups (Figure S9). The frequency of CD4^+^CD25^+^ non‐Tregs was significantly higher in the RAPA group than in the healthy controls and FK506 group. Compared with the healthy controls and RAPA group, the frequency of Tregs was the lowest in the FK506 group. Figure 2E summarized the statistical analyses of the healthy controls, FK506 and RAPA groups. Besides, suppressive proteins CTLA‐4 and lymphocyte activation gene‐3 (LAG3) were positive on all CD25^+^ clusters, including Tregs (Figure 1C). The frequency of the No.3 cluster, CD25^+^CTLA‐4^+^PD‐1^+^ (programmed cell death‐1) T_CM_, appeared twice as high in the RAPA group as in the FK506 group (Figures 1C and 2D). Distinct Tregs and suppressive protein expression on CD4^+^CD25^+^ T‐cells in the RAPA group suggest stabilization of immune system with reactivation potential.^25^, ^26^, ^27^ Reactivation potential of CD25^+^ T subpopulations, including Tregs and No.3 T_CM_, implied that T cells in the RAPA group could be better recruited in anti‐tumour or anti‐pathogen activities.

### 2.3 The suppressive proteins are enriched in T lineages with long‐term RAPA treatment after renal transplantation

**FIGURE 3:**
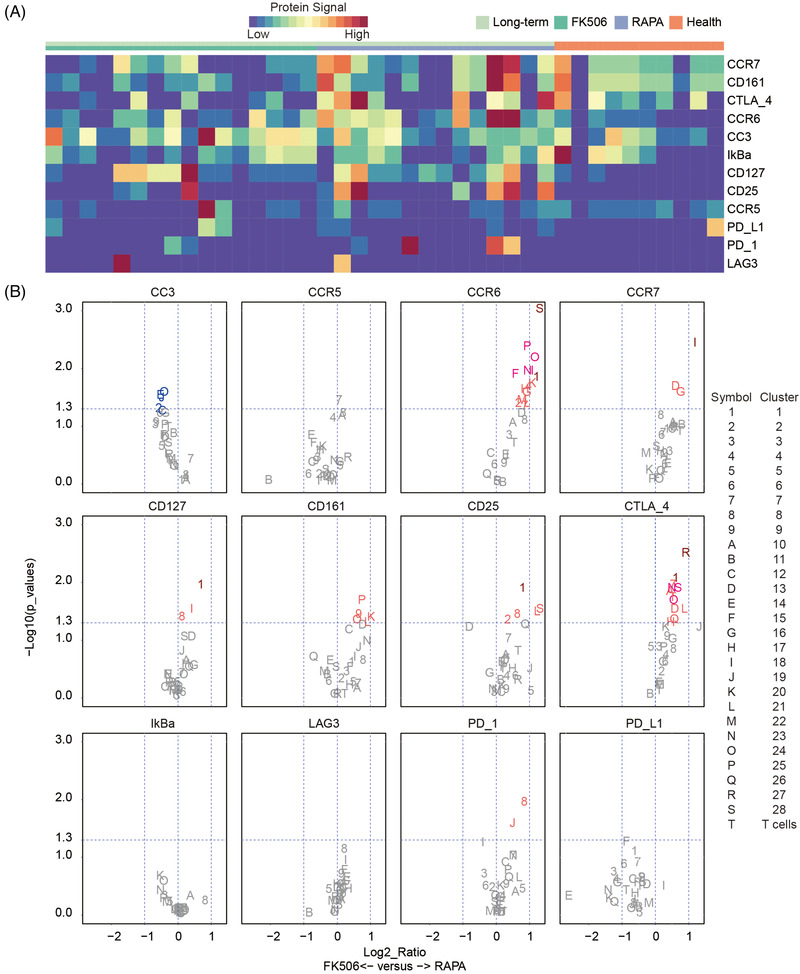
Health controls, FK506 and rapamycin (RAPA) groups expressed distinct functional proteins. (A) Heatmap representing the ArcSinh‐transformed expression values of functional proteins of all T cells in each individual. (B) Volcano plots of significantly differentially expressed functional proteins (Log_2_ Ratio ≠ 0 and −Log_10_ (*p*‐values) < 1.3 as adjusted *p* < 0.05) between the FK506 and RAPA groups. The symbol represents the cluster number and the letter T refers to the entire T‐cell lineage. Symbols of significant protein (*p* < 0.05) were coloured red (RAPA) or blue (FK506), and non‐significant functional proteins are coloured gray. The dark red and pink symbols respectively represent *q* < 0.5 and *q* < 0.1. All *p*‐values were calculated using unpaired Mann–Whitney test, and *q*‐values were corrected *p*‐values by Benjamini–Hochberg adjustment

The functional protein expression was further analyzed, and the heatmap (Figure 3A) depicts functional protein expression on individuals of healthy controls, FK506 and RAPA. The analyses of the functional protein expression were performed on 40 unstimulated samples. The comparison of functional protein expression between healthy controls and long‐term groups revealed that PD‐1, CCR6, CD25 (IL‐2 receptor) and CD127 (IL‐7 receptor) had higher expression levels in the long‐term groups (Figure S10). Compared with long‐term groups, CCR5, PD‐L1 and CTLA‐4 had higher expression levels in the health controls in over 10 clusters (Figure S10). The upregulations of activation and proliferation‐associated proteins, including CD25,^28^, ^29^ CD127 ^30^, ^31^, CD196,^32^, ^33^ on non‐Tregs in the long‐term groups may be caused by continuous stimulation of graft, which is involved in allograft rejection. The volcano plots were further performed to demonstrate distinct protein expression of the 28 clusters identified by PhenoGraph and the entire T‐cell lineage between the FK506 and RAPA groups (Figure 3B). Comparison of the FK506 and RAPA groups showed significant differences in the expression levels of CCR6, CCR7, CD127, CD25 and CTLA‐4 in some of the clusters, which is up to two‐fold higher in the RAPA group than in the FK506 group (Figure 3B and Figure S11).

### 2.4 The signaling pathway of TCR in the FK506 group is less responsive

**FIGURE 4:**
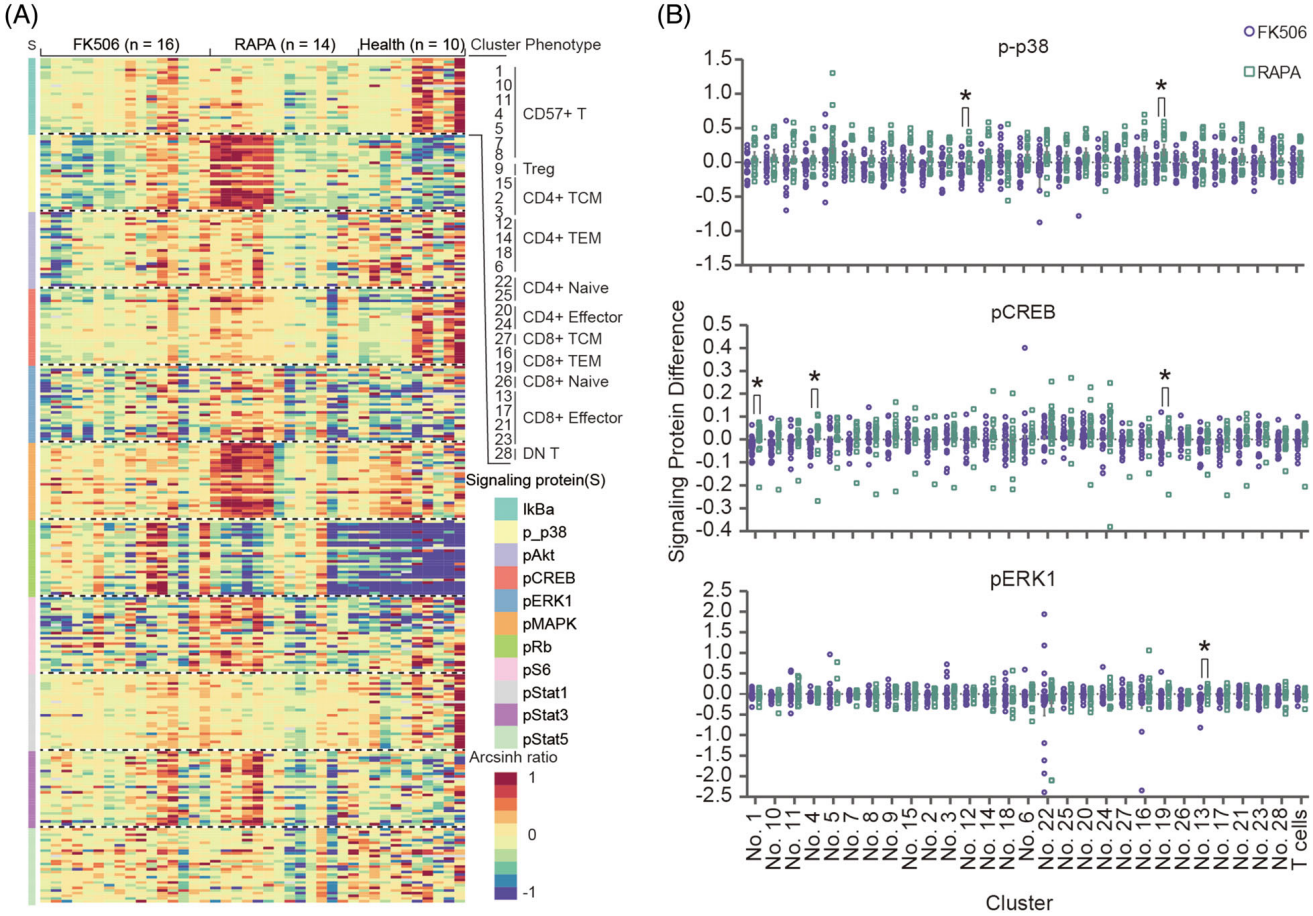
Signaling correlation clusters show less responsive T cell receptor (TCR) pathway in the FK506 group. (A) The heatmap depicts stimulus‐induced changes in 11 intracellular phosphorylation proteins in the 28 clusters across all tested groups. Each grid refers to the change in an intracellular signaling protein in a cluster, with colour indicating the ratio. The 308 rows consist of 11 signaling proteins multiplied by 28 clusters identified by PhenoGraph. Each of the 40 columns shows the signaling pathway variation profile of a tested sample. The sequences of the signaling proteins and clusters are separately annotated on the right. The colour scale indicates the logarithmically transformed difference between the ArcSinh‐transformed mean signal intensity in control and 2 min after stimulation, normalized per row by z‐score. (B) Signaling responses of p‐p38, pCREB and pERK1 significantly increased in T cell subpopulations of the rapamycin (RAPA) group compared to that in the FK506 group (*q* < 0.05). All *q*‐values were corrected *p*‐values by Benjamini–Hochberg adjustment, and *p*‐values were calculated using unpaired Mann–Whitney test

Next, we sought to explore the difference of signaling pathways between healthy controls and FK506 or RAPA groups in each subpopulation by analyzing their distinct signaling pathway of TCR through CD3/CD28 stimulation. Heatmap depicts CD3/CD28‐induced changes in the intracellular signaling responses of 29 different cell clusters, including 28 clusters identified by PhenoGraph and the entire T‐cell lineage. Twenty‐eight clusters were categorized into 11 major phenotypic subpopulations which are consistent with cluster classifications in Figure 1B‐D. The changes in 11 intracellular phosphorylation proteins in 28 clusters were calculated among the samples from the healthy controls and long‐term groups (Figure 4A). Compared with healthy controls, the expressions of pRb, p‐p38, pAkt and IκBα changed differently after CD3/CD28 stimulation in the long‐term groups (Figure 4A and Figure S12). As reported in the literature, degradation of IκBα, an inhibitor of the NF‐κB signaling pathway, indicates the production of immune responding cytokines and chemokines.^34^ The RB, ERK1, p38, CREB and S6, signaling pathways downstream of the TCR, regulate the IκBα module by their phosphorylation along the signaling cascade.^35^ Compared with FK506, enhanced phosphorylation of ERK1, p38 and CREB was more significantly in effector or T_EM_ lineages of the RAPA group after stimulation, even though the changes of IκBα expression level after CD3/CD28 stimulation were not significantly different in the long‐term groups (Figure 4B and Figure S13). This phenomenon suggests that the TCR signaling pathway is more sensitive in the RAPA group than in FK506. Effector and T_EM_ lineages in the RAPA group retained a more susceptible ability to be activated.^36^

### 2.5 Validation for the disparity in percentages of Tregs and CD8^+^CD57^+^ T cells between the FK506 and RAPA groups

**FIGURE 5:**
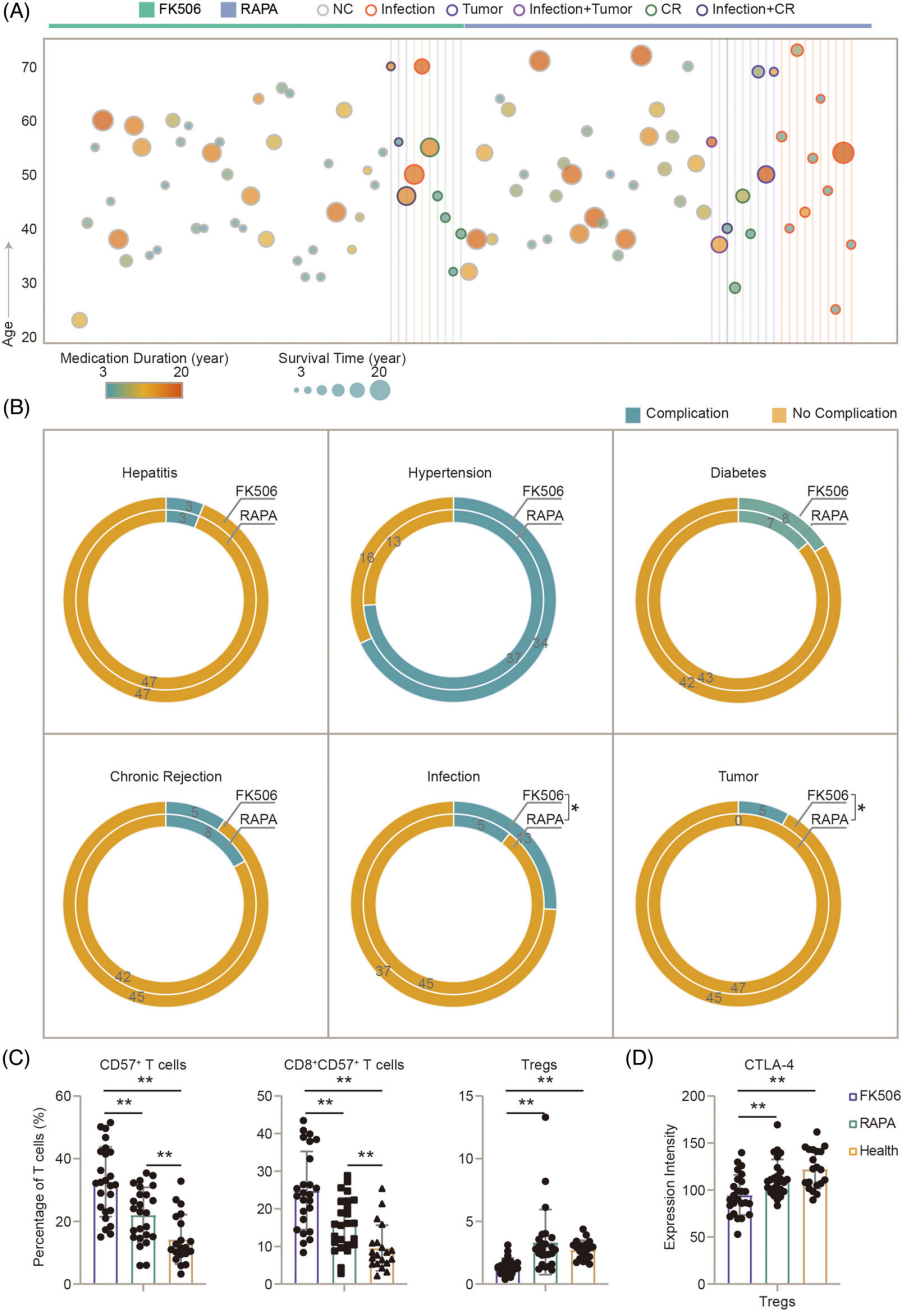
A validation cohort of 100 patients presents less tumour and infection in the long‐term rapamycin (RAPA) regimen treatment group. (A) The heatmap depicts the basic clinical information of 50 patients in the RAPA group and 50 patients in the FK506 group. The treatment duration of these 100 patients receiving renal transplantation in each group is more than 3 years without acute rejection. Clinical information included age, survival time after renal transplantation, duration of immunosuppressants treatment and diagnosis of infection, tumour and chronic rejection (CR). (B) The number of hepatitis, hypertension, diabetes, tumour, CR and infection in the FK506 (*n* = 50) and RAPA (*n* = 50) groups were counted and compared. *q < 0.05. Statistical analysis was performed using chi‐square test. (C) Percentages of CD57^+^, CD8^+^CD57^+^ T cells and Tregs in total T cell were compared among the health controls (*n* = 20), FK506 (*n* = 25) and RAPA (*n* = 25) groups. (D) Expressions of CTLA‐4 in Tregs were compared among the healthy controls, FK506 and RAPA groups. Statistical analysis in (C) and (D) was performed using unpaired Mann–Whitney test and Benjamini–Hochberg adjustment. Error bars indicate mean ± s.e.m. **q < 0.01

To further clarify the chronic influence of FK506 or RAPA on renal recipients, we then performed statistics on the incidence of the tumour, chronic rejection and infection during consistent treatment of immunosuppressants in the FK506 and RAPA groups (Table S2). Clinical data of an independent cohort with 100 randomly selected patients receiving renal transplantation were recruited, as described in the *Method and Material* section (Table S2). These patients have been consistently taking FK506 or RAPA for more than 3 years without acute rejection (Figure 5A). We found that the incidence of infection in the RAPA group was half that of the FK506 group. Moreover, none of the RAPA group was diagnosed with tumour, suggesting that the RAPA group could be more resistant to infection and tumour than the FK506 group when both renal allograft functions of FK506 and RAPA groups are stable (Figure 5B). Since the triple immunosuppression regimen received by recipients in the FK506 and RAPA groups included mycophenolate mofetil (MMF) and steroids, we compared the doses of MMF and steroids between the FK506 and RAPA groups. Doses of MMF and steroids were not significantly different between the FK506 and RAPA groups (Table 1 and Table S2). The Pearson correlation method was performed to test the correlations between doses and functional protein expressions or differences of signaling pathway in T cells in the long‐term groups for the two drugs (MMF or steroids). All coefficients were below 0.7, further suggesting MMF and steroids are not a key factor in educating the immune system in FK506 or RAPA groups in this study (Figures S14‐S15).

The percentages of Tregs, CD57^+^, CD8^+^CD57^+^ T cells were further detected by fluorescent flow cytometry in an independent validation cohort, consisting of the healthy controls (*n* = 20), FK506 (*n* = 25) and RAPA (*n* = 25) groups. The 50 patient samples that received renal transplantation were randomly collected from the cohort of 100 patients recruited for clinical statistical analyses (Figure 5C and 5D). A panel of seven fluorescent monoclonal antibodies was designed to analyze the percentage of Tregs, CD57^+^ and CD8^+^CD57^+^ T cells (Figure S16 and Table S3). Compared with the healthy controls, the percentages of CD57^+^ and CD8^+^CD57^+^ T cells were significantly higher in the FK506 and RAPA groups. The percentages of CD57^+^ and CD8^+^CD57^+^ T cells were 25% higher in the FK506 group than in the RAPA group. Meanwhile, the percentage of Tregs in the FK506 group was half that of the RAPA group and healthy controls (Figure 5C). Furthermore, compared with the healthy controls and RAPA group, the expression level of CTLA‐4 was 10% lower in the FK506 group (Figure 5D). The comparison results in Figure 5, acquired by fluorescent flow cytometry, are in accordance with former results in the Figures 2 and 3.

## 3 DISCUSSION

Along with the extended survival time, many chronic complications after transplantation are closely related to the selection of different immunosuppressive regimens. Therefore, a comprehensive understanding of the immune system chronically influenced by FK506 or RAPA is essential. CyTOF using a single‐cell suspension was applied to investigate the phenotype and potential response of the immune cells after TCR stimulation in renal allograft recipients receiving either FK506 or RAPA for maintenance of long‐term immunosuppression.^16^, ^17^, ^18^ Through in‐depth CyTOF analyses, our results showed distinct suppressive immune‐patterns between these two regimens: CD8^+^CD57^+^ T cells and Tregs were respectively induced in the FK506 and RAPA groups, and suppressive proteins were regulated distinctly in the RAPA group, which was validated in an independent cohort by fluorescent flow cytometry. Our study further reveals a less responsive signaling pathway of TCR was induced in the FK506 group.

Chronic exposure to alloantigen and incomplete T cell activation associated with immunosppressants, such as FK506, RAPA, and MMF, are thought to be responsible for the suppressive immune‐patterns.^3^, ^22^, ^37^ Compared with other immunosuppressants including RAPA and MMF, the FK506 is more efficient on immune suppression, which can inhibit production of de novo donor‐specific antibodies and prolong the lifetime of renal graft.^38^, ^39^ Expression of CD57 is gradually accumulated in the T cells, especially in the CD8^+^ T‐cell, under chronic antigen exposure. Up‐regulation of CD57 on CD8^+^ T cells is defined as senescence, and senescent T cells are late differentiated and antigen‐specific oligoclonal, which represent severely shortened telomerase signature. Frequencies of senescent CD8^+^CD57^+^ T cells appeared significantly higher abundance in the FK506 group which is beneficial for the improvement of acceptance and functional stable of graft ^40^, ^41^ and reduction of the consumption to maintain iatrogenic immunosuppression.^22^ Recent studies have shown that CD8^+^CD57^+^ T cells have a great impact on cancer, chronic intracellular infections, some chronic pulmonary diseases, autoimmune diseases and allogeneic transplantation, due to immunosuppressive activity mediated by CD8^+^CD57^+^ T cells.^42^, ^43^, ^44^, ^45^, ^46^ The appearance of CD8^+^CD57^+^ T cells is associated with malignancy (lung cancer and pleural mesothelioma),^47^, ^48^, ^49^ chronic viral infections (human immunodeficiency virus, human cytomegalovirus, Epstein–Barr virus, and hepatitis C virus),^50^, ^51^, ^52^, ^53^, ^54^ mycobacterium infections (pulmonary tuberculosis),^50^, ^55^ autoimmune disease (multiple sclerosis,^56^ type 1 diabetes mellitus,^56^ and rheumatoid arthritis.^57^ CD8^+^CD57^+^ T cells may produce high levels of pro‐inflammatory cytokine tumour necrosis factor‐α (TNF‐α).^50^, ^58^ The dysregulated TNF‐α was reported to be related to oncogenesis and inflammatory process.^59^, ^60^, ^61^ Pathway of PD‐L1 and CTLA‐4 is in charge of maintaining the self‐tolerance and balance between stimulatory and inhibitory.^62^, ^63^ The decrease expression level of PD‐L1 and CTLA‐4 may indicate that the balance of negative regulations was changed after receiving kidney transplantation. Negative regulatory function induces a stable immune system but also is associated with a reduced potential for resistance to the risk of developing cancer, infection and autoimmune disease.

In the RAPA group, Tregs and expression of suppressive proteins, including CTLA‐4 and PD‐1, were significantly higher. Tregs were considered to be of great importance in immune homeostasis, guiding the research hotspot in transplantation.^64^, ^65^, ^66^ Previous research applied a preclinical renal transplantation model to simulate the cellular presensitization, a syndrome found in recipients after transplantation. Tregs, combined with CNI and exhaustion T cell, controlled donor reactive memory T cells, which is beneficial for withdrawal long‐term administration of CNIs.^64^ Up‐regulation of CTLA‐4 on Tregs activates the regulatory and suppressive function of Tregs.^63^, ^67^ Besides, the expression of CTLA‐4 on non‐Tregs may inhibit T cell function, which is beneficial for balancing the immune system under chronic antigen exposure in the RAPA group.^68^ Compared with the FK506 group, higher expression of CD127 ^30^, ^31^ in the RAPA group further implies the antimicrobial ability of RAPA, and the risk of allograft rejection is reduced in the FK506 group than in the RAPA group.^69^ Furthermore, the loss of T‐cell function is not completely irreversible, because T cells have the potential to be revived by blocking the pathway of PD‐1 and CTLA‐4. Both CD8^+^CD57^+^ T cells and Tregs are beneficial for maintaining immune system stable in FK506 and RAPA group, while distinct CD8^+^CD57^+^ T cells in the FK506 group suggest irreversible suppression. The appearance of Tregs and widely expression of suppressive proteins in the RAPA group imply the potential of anti‐tumour, or anti‐infection is retained in recipients educated by long‐term administration of RAPA.

Furthermore, CD3/CD28 induced disparity of intracellular immune response between the FK506 and RAPA groups indicated RAPA induced a more sensitive immune response after kidney transplantation. TCR signaling pathway regulates the expression of cytokines and chemokines through NF‐κB signaling modules and controls activation and proliferation of T cells. The silence of NF‐κB signaling modules after CD3/CD28 stimulation in the RAPA and FK506 groups suggests FK506 and RAPA stably and effectively suppressed T‐cell function. However, up‐regulation of TCR downstream pathway after CD3/CD28 stimulation in the RAPA group indicated the awakening capacity of T cells in RAPA group.^35^, ^36^ Future studies will focus on the formally functional capacity of CD8^+^CD57^+^ or CD4^+^CD25^+^ T‐cells emerging after transplantation.

CD8^+^CD57^+^ and Tregs play a distinct role in maintaining the stability of the immune system and suppressing T cell function in the FK506 and RAPA groups. When patients in both groups were stable, the suppressive function of CD8^+^CD57^+^ T cells in the FK506 group was more irreversible. Minor changes of TCR downstream pathway after CD3/CD28 stimulation in the FK506 group suggest complete suppression on T cells by FK506. The proportion of CD8^+^CD57^+^ T cells in the RAPA group was about half in the FK506 group, but the inadequacy of maintaining graft acceptance was compensated by Tregs and suppressive proteins. In the RAPA group, Tregs and suppressive proteins on conventional T cells played immuno‐regulatory roles. The negative regulatory function of Tregs and suppressive proteins is not completely irreversible in response to specific stimuli, implying T cells in the RAPA group are more likely to be reactivated to participate in new adaptive immunity when anti‐tumour or anti‐infection immune responses are concerned. Clinical data of independent 100 patients receiving renal transplantation indicates that oncogenesis and infection were indeed attenuated in the RAPA group when both immune systems of FK506 and RAPA groups are stable. Complications associated with prolonged survival should be considered in the selection of immunosuppressants. For example, RAPA may be more suitable for renal graft recipients with previous diagnosis of cancer, while FK506 for recipients with more robust immunity. Nevertheless, the assumptions need further experimental and clinical validation.

In conclusion, the major contributions from this study can be summarized below. (1) The distinct immune landscape and T cell lineage regulatory patterns educated by long‐term treatment of FK506 and RAPA have been depicted in this study. Senescent CD8^+^CD57^+^ T lineages and Tregs respectively increased in the FK506 and RAPA groups. (2) This study reveals the difference in deep phenotyping of T cells between the FK506 and RAPA groups, suggesting a discrepancy in the reactivation potential of the two groups when an anti‐tumour or anti‐infection immune response is concerned. The functional capacity of Tregs and suppressive proteins in the RAPA group indicates the potential to recover T lineages retained in the RAPA group. Meanwhile, the increased proportion of CD8^+^CD57^+^ T lineages induced by FK506 and less responsive T‐lineages in the FK506 group indicates better graft acceptance. (3) This study provided new insights into the anti‐tumour and anti‐infection potential of the immune system educated by RAPA. A validation cohort of independent 100 patients was recruited for the hypothesis verification that the RAPA regimen manifests less oncogenesis and infection during long‐term administration of immunosuppressants. In essence, this study provides a fundamental understanding of the immune system chronically induced by FK506 or RAPA, and, most of all, it is helpful for rationale and perspective evidence‐based guidance for long‐term personalized medicine.

## 4 METHODS AND MATERIALS

### 4.1 Patients

This study was approved by the Ethics Committees of the Ruijin Hospital, Shanghai Jiaotong University School of Medicine. The clinical trial registry number is *KY2018‐153*, and written informed consents were obtained from all recipients. A discovery cohort included 30 and the independent cohort recruited 100 first renal transplant recipients who received an FK506‐based, or a RAPA‐based regimen for at least 3 years. All participants received a living‐related donor transplant between 1995 and 2018. A triple immunosuppression regimen consisting of FK506 or RAPA, MMF and steroids were administered to the 130 recipients. Doses of MMF and steroids were no significant difference between FK506 and RAPA groups (Table 1 and Table S2). The allograft functions were stable, and there was no episode of allograft rejection. Among them, 66 patients received FK506 at a blood concentration of 5–7 ng/ml, and 64 renal allograft recipients received RAPA at a blood concentration of 3–5 ng/ml. MMF was administered between 1000–1500 mg daily. The steroid regimen was 5–7.5 mg daily. The demographic data between the two groups were comparable. All the patients’ follow‐up records during the 3‐year study are documented and available as supplementary materials. Thirty age‐ and sex‐matching healthy controls were recruited from the Ruijin Hospital, Shanghai Jiaotong University School of Medicine. Before being enrolled in our study, written informed consent was collected from renal transplant recipients and healthy controls.

### 4.2 Cell preparation and stimulation

To study immune‐phenotype and phosphorylation events, lymphocytes extracted from peripheral blood of 30 patients in the discovery cohort were used. Peripheral blood with trough concentrations of immunosuppressants was collected at half an hour before taking the medicine. After extraction of lymphocytes, cells were resuspended in DMEM (Thermo Fisher Scientific) with 5μM cisplatin (Sigma‐Aldrich). After cisplatin treatment, cells were assigned to the control group or stimulation group. The control group was set as the baseline for phosphorylation events. The stimulation group was stimulated with 6 μg/ml CD3e (Biolegend) and CD28 (Biolegend). Stimulation occurred in a plate coated with 5 μg/ml CD3e, and cells were incubated for 2 min at 37°C. After stimulation, cell activation was stopped by the addition of paraformaldehyde with a final concentration of 1.6% (Sangon Biotech). PFA‐fixed lymphocytes were stored at −80°C. The cryoprotectant was consisting of 10% DMSO (Adamas‐beta) and 90% Maxpar cell‐staining buffer (Fluidigm Sciences).

### 4.3 Antibody preparation

Antibodies, manufacturers and the concentrations used in this study are listed in Table S3. Pre‐labelling metal‐conjugated antibodies were purchased from Fluidigm Sciences. Purified antibodies (Table S3) were labelled by using the Maxpar × 10 antibody labelling kit (Fluidigm Sciences). After conjugation, antibodies were stored at 4°C.

### 4.4 Immunostaining

Surface and intracellular signaling proteins were stained. The 80 samples included two samples per person in all the FK506, RAPA and Health groups, which were either stimulated by CD3/CD28 or not treated (control). Frozen lymphocyte suspensions were thawed on ice. Three million cells per sample were stained in 100 μl final volume. Cells were blocked with Fc blocking solution (BioLegend) and then stained with a cocktail of 17 metal isotope conjugated antibodies against surface proteins (Table S3), including CD3, CD4, CD8a, CD25, CD45, CD45RA, CD57, CD127, CD161, CD45RO, CCR5, CCR6, CCR7, PD‐L1, PD‐1, CTLA_4 and LAG3. After staining of surface proteins, cells were treated with methanol and then stained with 12 antibodies against intracellular signaling proteins (Table S3), including caspase 3, pStat1, pStat3, pStat5, p‐p38, pERK1/2, pS6, pCREB, pMAPKAPK II, IκBα, pRB and pAKT. Cells were then stained for 30 min at room temperature with 100 μl of 191Ir,193Ir DNA intercalator (Fluidigm Sciences) diluted 1:4000 in phosphate buffer saline (PBS).

### 4.5 Data acquisition and post‐processing

Data were acquired by Helios (DVS Sciences) with previously described instrument settings.^15^ To minimize batch‐to‐batch variance, a standard internal metal isotope bead was acquired with samples together as a normalization guideline.^15^

### 4.6 Flow cytometry

To validate the result acquired by CyTOF, the 25 patient samples in the FK506 and 25 patient samples in the RAPA group were randomly collected from the independent cohort of 100 renal transplantation patients recruited for clinical statistical analyses. Fluorescent flow cytometry was performed on the validation cohort, including 50 renal transplantation patients and 20 age‐ and sex‐matching healthy controls, and a panel of seven surface antibodies was detected (Table S3). Peripheral blood was treated for 15 min at room temperature with 200 μl of Lysing Buffer (BD Pharmingen) diluted 1:9 in PBS and then washed twice with phosphate buffer saline (PBS). Cells were resuspended in 100 μl PBS and blocked with Fc blocking solution (BD Pharmingen) for 10 min at room temperature. Blocked cells were stained for 15 min at room temperature with 100 μl of BD Horizon Fixable Viability Stain (Fluidigm Sciences, San Francisco, USA) diluted 1:1000 in PBS and then stained with a cocktail of seven antibodies, including CD3, CD4, CD8, CD25, CD57, CD127 and CTLA‐4, for 30 min at 2–8°C. Then samples were resuspended in 400 μl PBS and analyzed by flow cytometry in a Celesta (BD Biosciences). Cytobank was employed for analyzing acquired 70 standard CyTOF data files (FCS files), including 25 FK506 datasets, 25 RAPA datasets and 20 health datasets.

### 4.7 Data analysis

#### 4.7.1 High dimensional data analysis

Acquired 80 standard format for data files of flow cytometry and mass cytometry (FCS) files, including 32 FK506 datasets, 28 RAPA datasets and 20 health datasets, were uploaded into Cytobank, and a series of gates were used to select single and living T (CD45^+^CD3^+^) cells, as depicted in Figure S2. T cells were exported from all acquired events as new FCS files for further studying by Cytofkit. 1 × 10^4^ cells were extracted from data pooled from the PBMC samples randomly through down‐sampling. CytofAsinh was performed for transforming signal intensities of each channel with a cofactor of 5. Clustering was carried out by two steps: First, FlowSOM was used to cluster all the cells into 2500 nodes, and then all the node centers were clustered to metacluster by PhenoGraph. FlowSOM analysis was applied to cluster analysis using the FlowSOM package in R, and PhenoGraph analysis was used for meta‐clustering analysis. Two thousand events were selected randomly from each sample, and the t‐SNE was used for visualization. Unstimulated samples were applied to analyses of phenotype in Figure 2 and functional protein expression in Figure 3.

#### 4.7.2 Intracellular protein analysis

The heatmap depicts changes of each intracellular protein, cell subset and group using the pheatmap R package (v. 1.0.12) (Figure 4). The change of each intracellular protein was calculated as the logarithmically transformed difference between the ArcSinh‐transformed mean signal intensity in control and 2 min after stimulation. The colour scale indicates a logarithmically transformed difference of the signaling responses, normalized to (−1, 1).

#### 4.7.3 Volcano plots

Functional protein was visualized by a volcano plot using R package SPADEVIzR.^70^ Volcano plots present differentially expressed functional proteins comparing FK506 and RAPA groups.

#### 4.7.4 Statistical analysis and visualization

Statistical analysis was performed using GraphPad Prism 8 and R 3.6.1. Group comparisons in Figures 2–4 and 5C were performed using unpaired Mann–Whitney test with Benjamini–Hochberg adjustment. Group comparisons in Figure 5B were performed using chi‐square test.

## CONFLICT OF INTEREST

The authors declare that they have no conflict of interest.

## Supporting information

Supporting information
