# Supplementary material for: Deep phenotyping of T cell populations under long‐term treatment of tacrolimus and rapamycin in patients receiving renal transplantations by mass cytometry
Source: Clin Transl Med. 2021 Nov 8;11(11):e629. doi: 10.1002/ctm2.629 (PMC8574956; doi:10.1002/ctm2.629)
Supplement: Supplementary file 1 — Supporting information [file CTM2-11-e629-s001.docx]

**Supplementary Materials**

**Table S1 The correspondence between 28 clusters and identified 18 phenotypical subpopulations**

| Phenotypic subpopulation | The 28 clusters identified by PhenoGraph | | | | | | | | | | | |
| --- | --- | --- | --- | --- | --- | --- | --- | --- | --- | --- | --- | --- |
| CD57^+^ T cell | No.1 | No.4 | No.5 | No.7 | No.8 | No.10 | No.11 |  |  |  |  |  |
| CD4^+^CD57^+^ T cell | No.4 |  |  |  |  |  |  |  |  |  |  |  |
| CD8^+^CD57^+^ T cell | No.5 | No.7 | No.8 | No.10 | No.11 |  |  |  |  |  |  |  |
| DN CD57^+^ T cell | No.1 |  |  |  |  |  |  |  |  |  |  |  |
| CD4^+^CD57^-^ T cell | No.2 | No.3 | No.6 | No.9 | No.12 | No.14 | No.15 | No.18 | No.20 | No.22 | No.24 | No.25 |
| CD4^+^ effector | No.20 | No.24 |  |  |  |  |  |  |  |  |  |  |
| CD4^+^ naïve | No.22 | No.25 |  |  |  |  |  |  |  |  |  |  |
| CD4^+^ T_EM_ | No.6 | No.12 | No.14 | No.18 |  |  |  |  |  |  |  |  |
| CD4^+^ T_CM_ | No.2 | No.3 | No.15 |  |  |  |  |  |  |  |  |  |
| Tregs | No.9 |  |  |  |  |  |  |  |  |  |  |  |
| CD8^+^CD57^-^ T cell | No.13 | No.16 | No.17 | No.19 | No.21 | No.23 | No.26 | No.27 |  |  |  |  |
| CD8^+^ effector | No.13 | No.17 | No.21 | No.23 |  |  |  |  |  |  |  |  |
| CD8^+^ naïve | No.26 |  |  |  |  |  |  |  |  |  |  |  |
| CD8^+^ T_EM_ | No.16 | No.19 |  |  |  |  |  |  |  |  |  |  |
| CD8^+^ T_CM_ | No.27 |  |  |  |  |  |  |  |  |  |  |  |
| DN T cell | No.28 |  |  |  |  |  |  |  |  |  |  |  |
| CD4^+^CD25^+^ T cell | No.3 | No.9 | No.25 |  |  |  |  |  |  |  |  |  |
| CD4^+^CD25^+^ CD127^+^ T cell | No.3 | No.25 |  |  |  |  |  |  |  |  |  |  |

**Table S2 Clinical information of the 109 patients in the validation cohort.**

| Characteristics of the sample | | | |  |
| --- | --- | --- | --- | --- |
| Demographics^*^ | | | |  |
| Gender (male/female) | FK506 | 32/18 | |  |
|  | RAPA | 25/25 | |  |
| Age (years) | FK506 | 49 (25-73) | |  |
|  | RAPA | 48 (23-70) | |  |
| Survival time after surgery (years) | FK506 | 8 (3-18) | |  |
|  | RAPA | 8 (3-21) | |  |
| Immunosuppressor use^†^ | | | |  |
| \| DSurvival time after surgery (years) \| FK506 \| 8 (3-18) \| \| --- \| --- \| --- \| \| RAPA \| 8 (3-21) \| | | | |  |
| Immunosuppressor (mg/day) | FK506 | MMF | 1000-1500  ns      ns |  |
|  |  | Steroid | 5-7.5 |  |
|  |  | FK506 | 3.5 (1-5) |  |
|  | RAPA | MMF | 1000-1500 |  |
|  |  | Steroid | 5-7.5 |  |
|  |  | RAPA | 0.83 (0.25-2) |  |
| *Values indicate number of patients or average and range.  †Milligram equivalent of oral tacrolimus or rapamycin respectively.Dose is average of cumulative dose per day. | | | |  |

**Table S3 Antibody list.**

| Mass cytometry antibodies | | | | | |
| --- | --- | --- | --- | --- | --- |
| Antigen | Mass Tag | Clone | Vendor | Cat# | Conc. |
| CD45 | ^89^Y | HI30 | Fluidigm | 3089003B | 1:100 |
| CCR6/CD196 | ^141^Pr | 9C4 | DVS Science | 3141006B | 1:100 |
| CD45RA | ^143^Nd | HI100 | Fluidigm | 3143006B | 1:100 |
| CCR5/CD195 | ^144^Nd | NP-6G4 | DVS Science | 3144007A | 1:100 |
| CD4 | ^145^Nd | RPA-T4 | Fluidigm | 3145001B | 1:100 |
| CD8a | ^146^Nd | RPA-T8 | Fluidigm | 3146001B | 1:100 |
| PD-L1/CD274 | ^148^Nd | 29E.2A3 | Fluidigm | 3148017B | 1:100 |
| CD45RO | ^149^Sm | UCHL 1 | Fluidigm | 3149001B | 1:100 |
| PD-1/CD279 | ^155^Gd | EH12.2H7 | Fluidigm | 3155009B | 1:100 |
| CD161 | ^160^Gd | HP-3G10 | Biolegend | 339902 | 1:50 |
| CTLA_4/CD152 | ^161^Dy | 14D3 | Fluidigm | 3161004B | 1:100 |
| LAG3/CD223 | ^165^Ho | 874501 | Fluidigm | 3165028B | 1:100 |
| CCR7/CD197 | ^167^Er | G043H7 | Fluidigm | 3167009A | 1:50 |
| CD127 | ^168^Er | A019D5 | Fluidigm | 3168017B | 1:100 |
| CD25 | ^169^Tm | 2A3 | Fluidigm | 3169003B | 1:100 |
| CD3 | ^170^Er | SP34-2 | Fluidigm | 3170007B | 1:100 |
| CD57 | ^172^Yb | HCD57 | DVS Science | 3172009B | 1:100 |
| Caspase 3 (Cleaved) | ^142^Nd | D3E9 | Fluidigm | 3142004A | 1:100 |
| pStat5 [Y694] | ^147^Sm | 47 | Fluidigm | 3147012A | 1:100 |
| pRb [S807/811] | ^150^Nd | J112-906 | Fluidigm | 3150013A | 1:100 |
| pAkt [S473] | ^152^Sm | D9E | Fluidigm | 3152005A | 1:100 |
| pStat1 [Y701] | ^153^Eu | 4a | Fluidigm | 3153005A | 1:100 |
| p-p38 [T180/Y182] | ^156^Gd | D3F9 | Fluidigm | 3156002A | 1:100 |
| pStat3 [Y705] | ^158^Gd | 4 | Fluidigm | 3158005A | 1:100 |
| pMAPKAPKII [T334] | ^159^Tb | 27B7 | Fluidigm | 3159010A | 1:100 |
| IkBa | ^164^Dy | L35A5 | Fluidigm | 3164004A | 1:100 |
| pERK 1/2 [T202/Y204] | ^171^Yb | D13.14.4E | Fluidigm | 3171010A | 1:100 |
| pS6 [S235/S236] | ^175^Lu | N7-548 | Fluidigm | 3175009A | 1:100 |
| pCREB [S133] | ^176^Yb | 87G3 | Fluidigm | 3176005A | 1:100 |
| Flow cytometry antibodies | | | | | |
| Antigen | Fluorescent Tag | Clone | Vendor | Cat# | Conc. |
| CD3 | FITC | UCHT1 | BD Pharmingen | 555332 | 1:5 |
| CD4 | PerCP-Cy™5.5 | RPA-T4 | BD Pharmingen | 560650 | 1:20 |
| CD8a | Alexa 700 | RPA-T8 | BD Pharmingen | 557945 | 1:20 |
| CD25 | PE | M-A251 | BD Pharmingen | 555432 | 1:5 |
| CD127 | BV421 | HIL-7R-M21 | BD Pharmingen | 562436 | 1:20 |
| CTLA_4/CD152 | Brilliant Violet 605 | BNI3 | Biolegend | 369610 | 1:20 |
| CD57 | PE-Cyanine7 | TBO1 | eBioscience | 25-0577-42 | 1:20 |

**
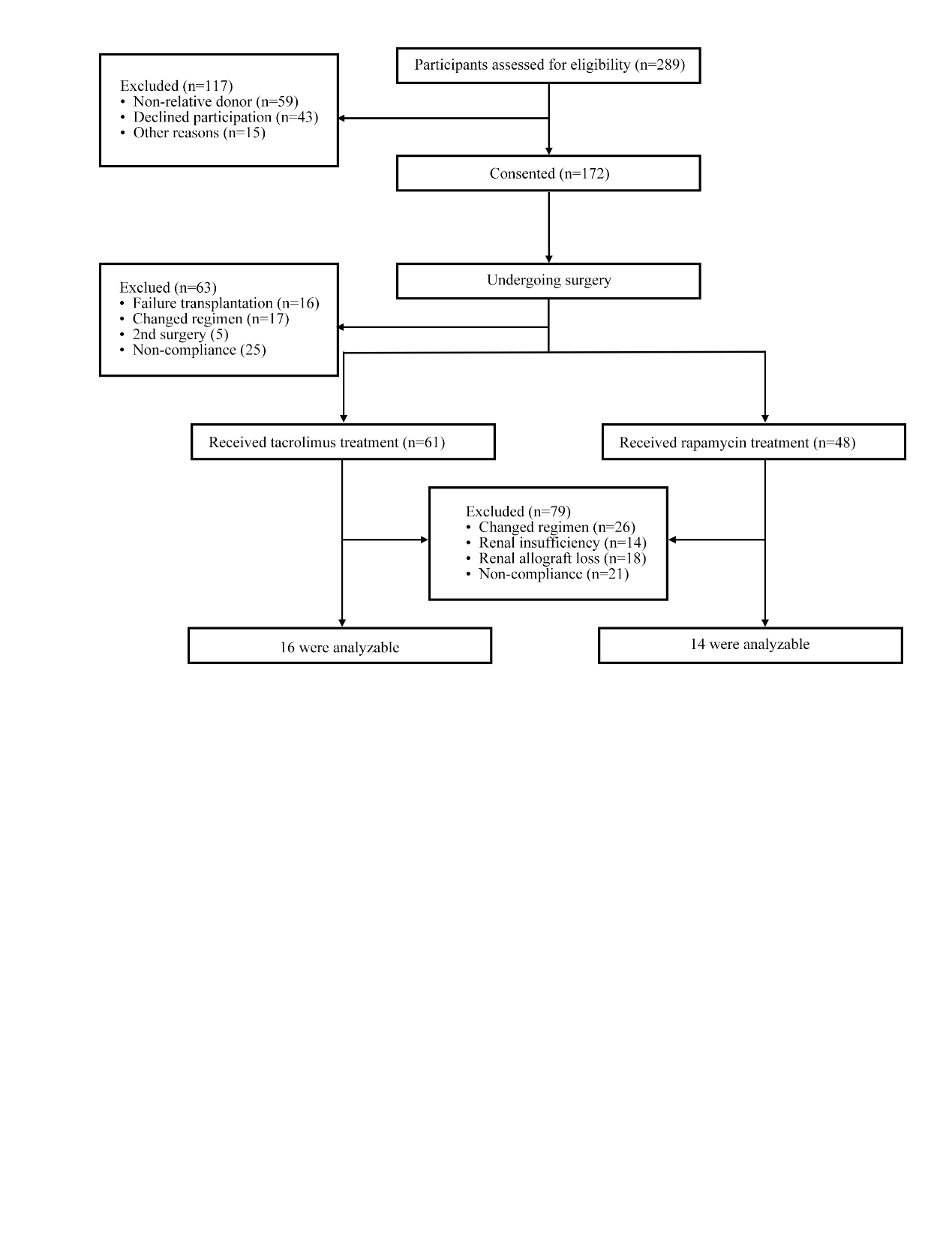
**

**Figure S1. Recruitment summary of 30 Patients in the discovery cohort.** In total, 289 patients were evaluated for eligibility; 172 patients consented, 109 patients received an FK506-based or a RAPA-based treatment after successful kidney transplantation, and 30 patients completed the study. The 30 patients included 16 patients in the FK506 group and 14 patients in the RAPA group.

**
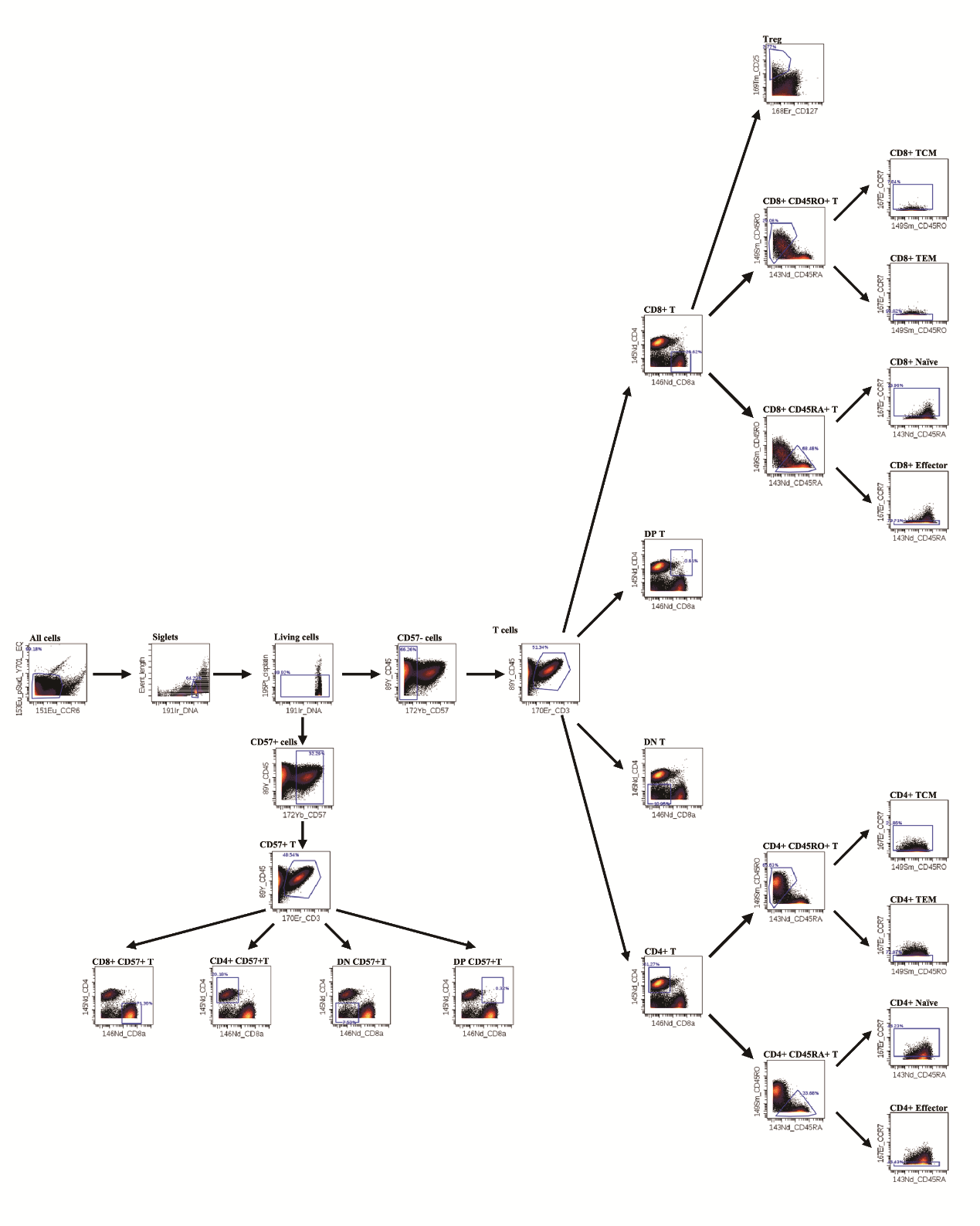
**

**Figure S2. Manual gating scheme.** A typical gating scheme was used to define classical immune cell types. A representative sample was used to display the gating scheme.T_EM_: effector memory T cells, T_CM_: central memory T cells, DP T: CD4^+^CD8^+^ T cells, DN T: CD4^-^CD8^-^ T cells.


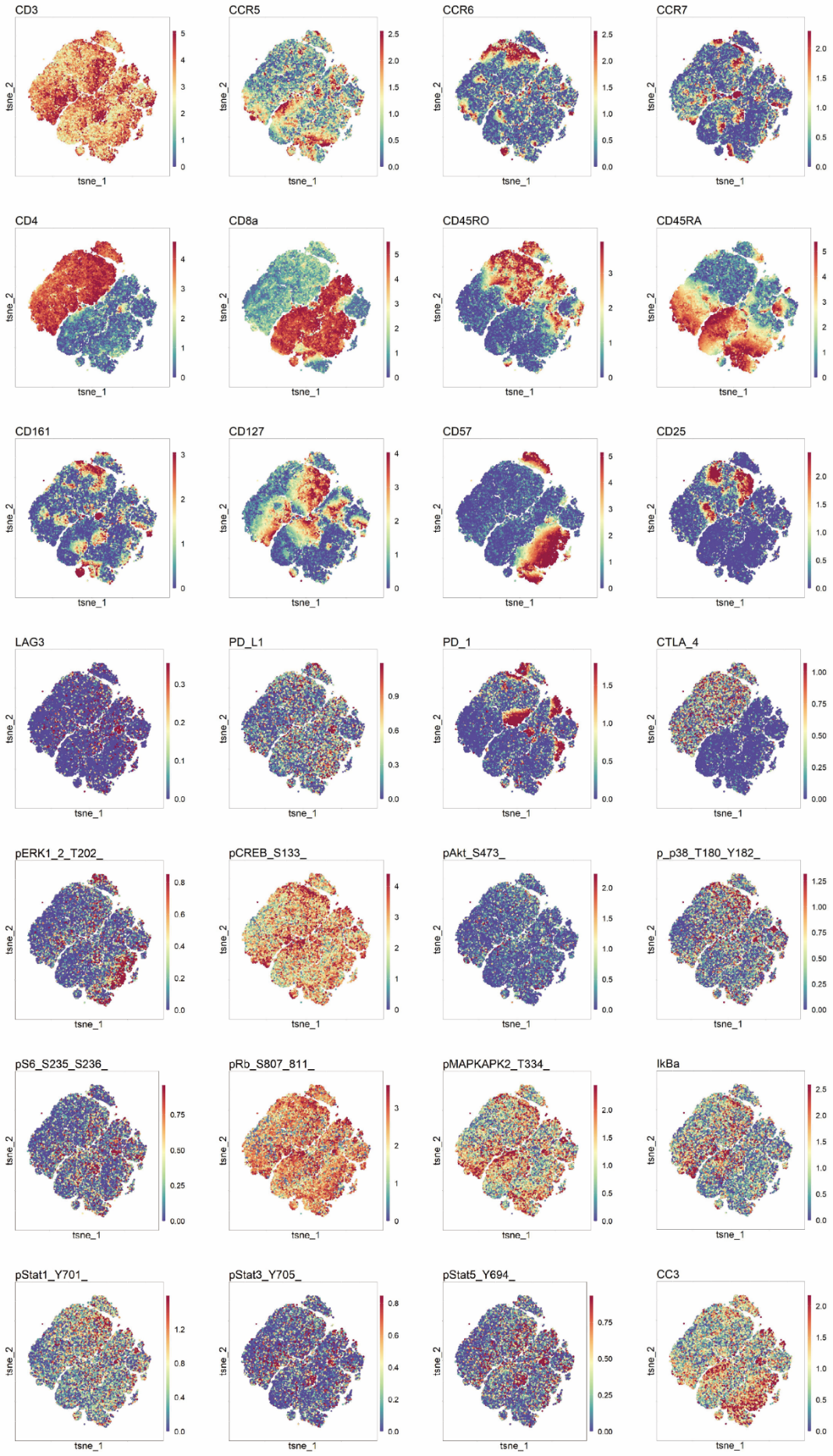


**Figure S3. t-SNE visualization of 28 proteins.** t-SNE visualization of T cells depicts the expression of 17 surface proteins and 14 signaling proteins. Colors represent the Asinh-transformed expression values of the indicated markers.


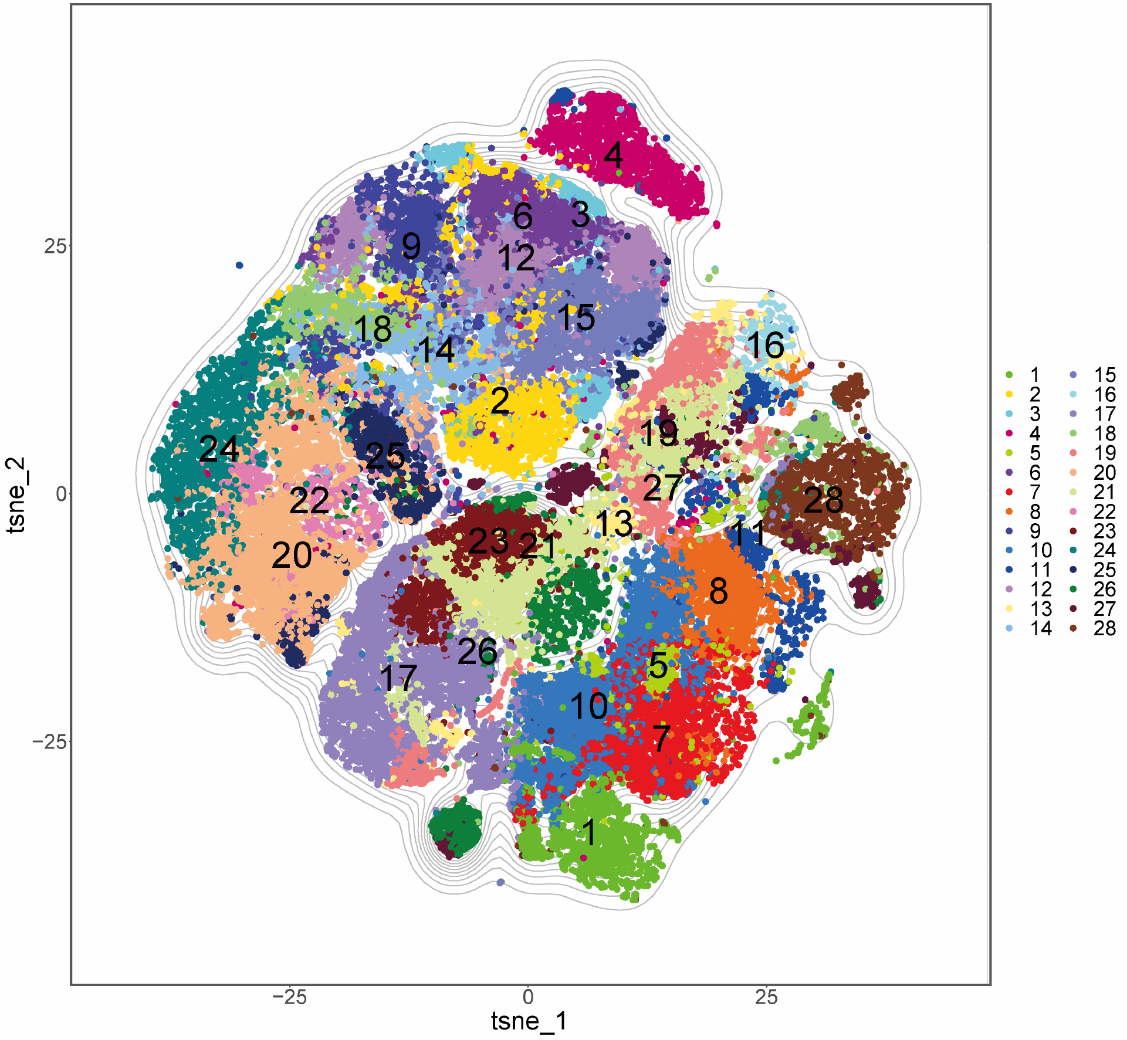


**Figure S4. 28 metaclusters identified by Phenograph.** FlowSOM and PhenoGraph were applied to cluster all the cells and 28 metaclusters were identified by PhenoGraph.12 surface proteins, including CD4, CD8, CD45RA, CD45RO, CCR5, CCR6, CCR7, CD127, CD25, CD57, CD161, and PD-1 were used in clustering analysis.

**
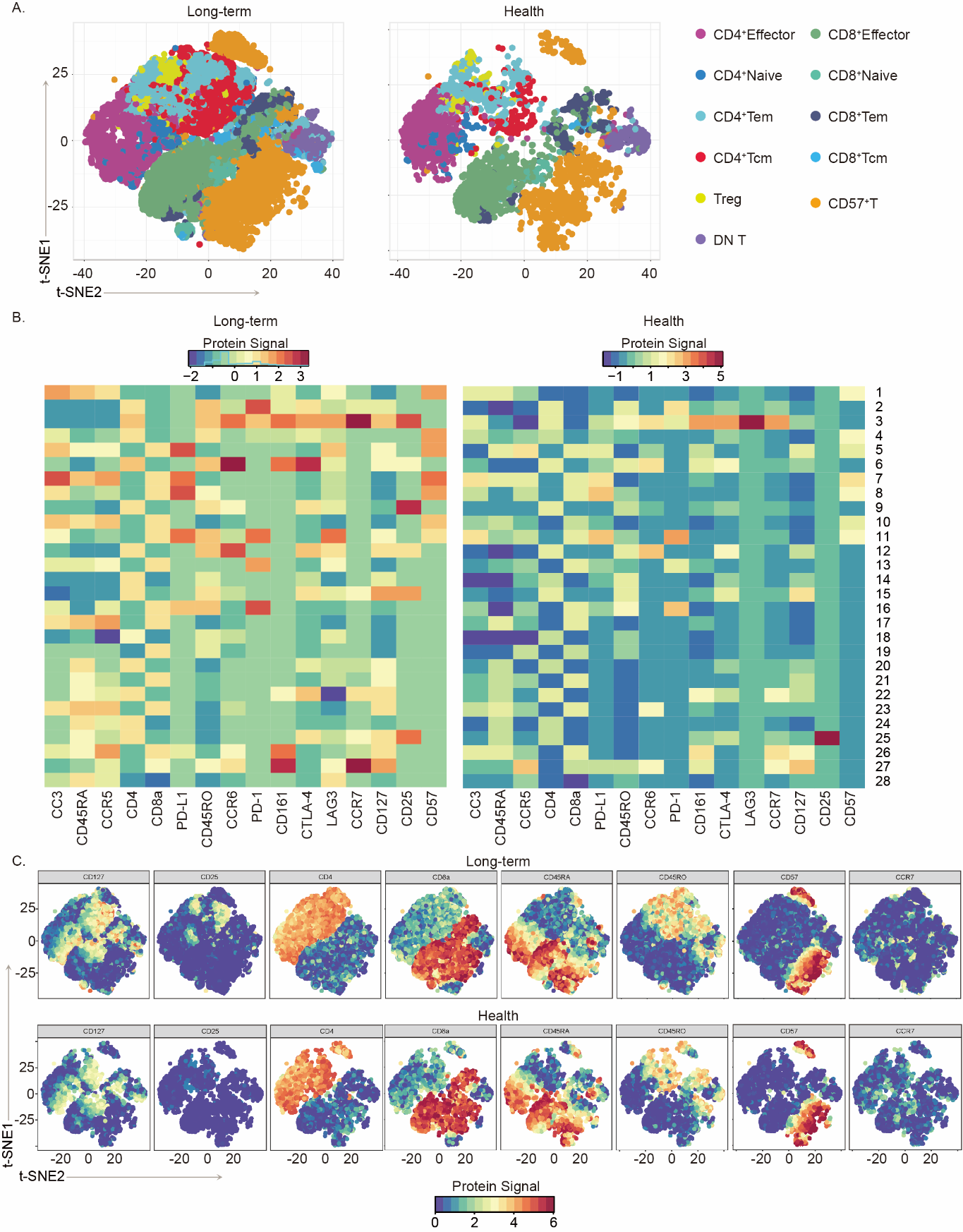
**

Figure S5. **t-SNE visualization of the healthy controls and long-term groups. (**A**)** t-SNE visualization of the long-term groups and healthy controls with labeled cluster partitions. **(B)** Heatmap shows mean expression values of surface proteins, normalized per column by z-score, in the long-term groups and healthy controls. **(C)** t-SNE visualization illustrating the expression of eight proteins used for identifying subpopulations in the long-term groups and healthy controls. Color in **(B)** and **(C)** represent the ArcSinh-transformed signal intensity of the indicated markers.

**
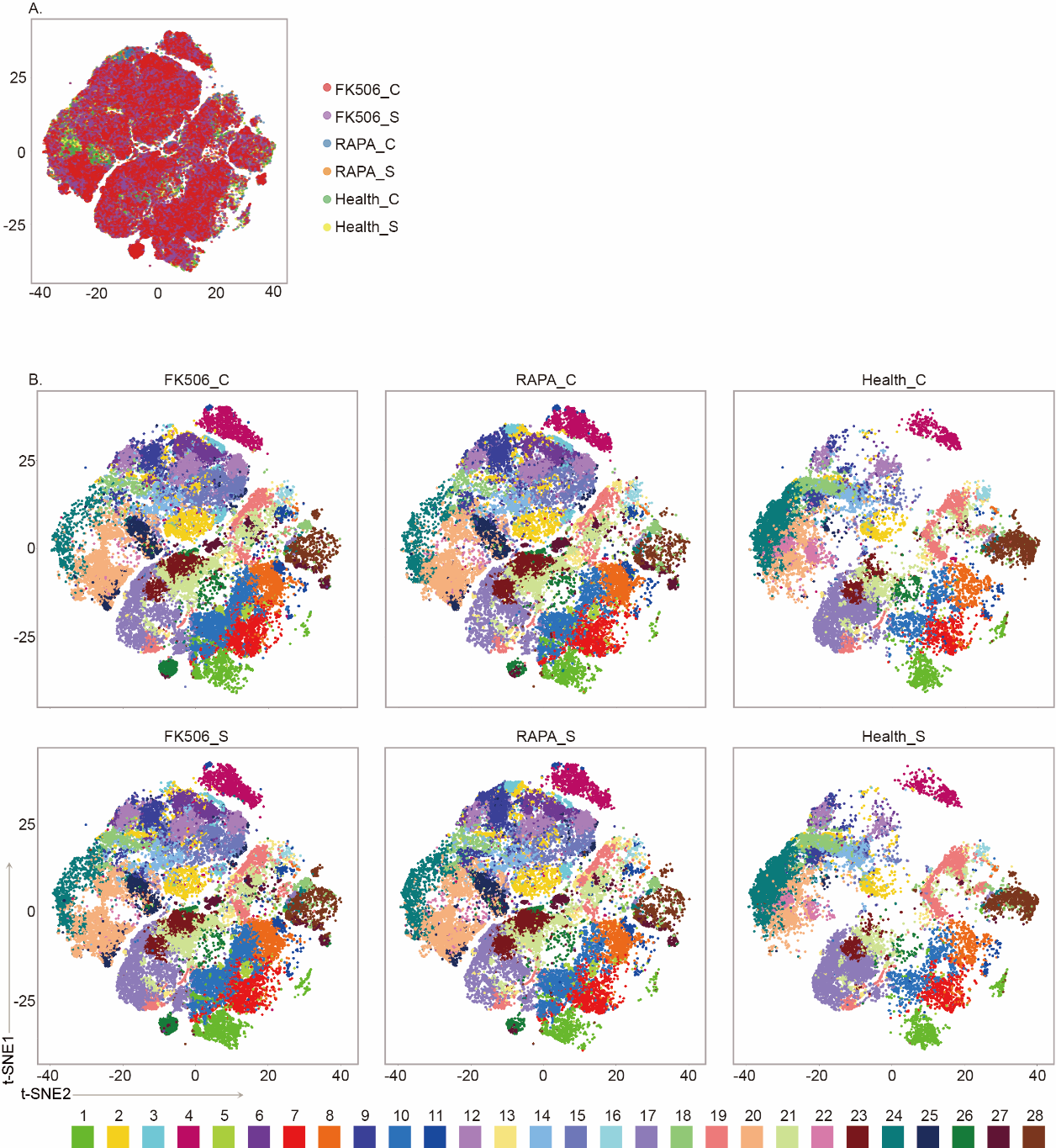
 Figure S6.t-SNE visualization of 6 groups. (A)** t-SNE presenting all T single-cell data from the healthy controls, the FK506 and RAPA groups was plotted through dimensionality reduction. The colors indicate the six sample groups: the healthy controls without stimulation (Health_C), the healthy controls with CD3/CD28 stimulation (Health_S), the FK506 group without stimulation (FK506_C), the FK506 group with CD3/CD28 stimulation (FK506_S), the RAPA group without stimulation (RAPA_C), and the RAPA group with CD3/CD28 stimulation (RAPA_S). **(B)** t-SNE maps of Health_C, Health_S, FK506_C, FK506_S, RAPA_C, and RAPA_S were presented separately.


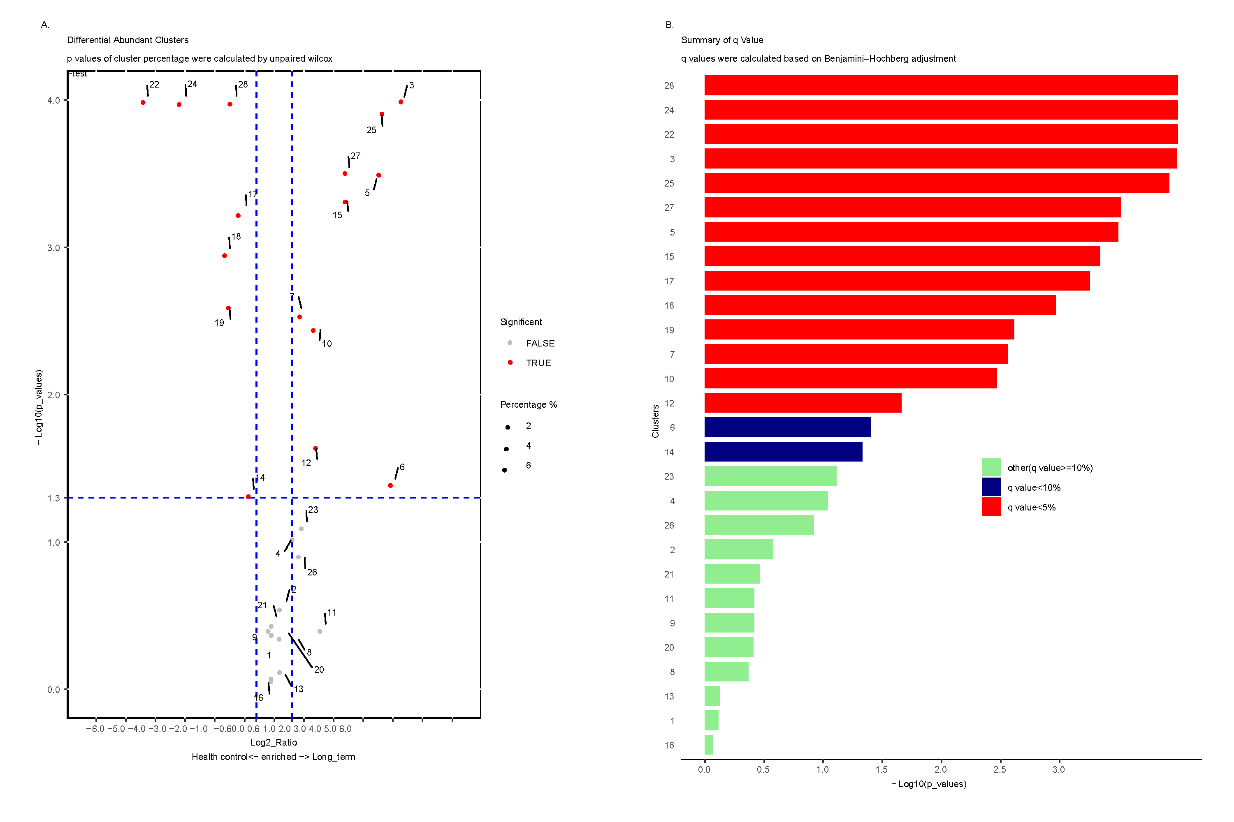
**Figure S7. Comparison of the long-term groups and healthy controls in frequencies of the identified 28 clusters. (A)** The volcano plot showed differentially abundant clusters, colored by red, between long-term groups and healthy controls. The symbol represents the cluster number. **(B)** Group comparisons between the long-term groups and healthy controls were performed using unpaired Mann-Whitney test with Benjamini-Hochberg adjustment.


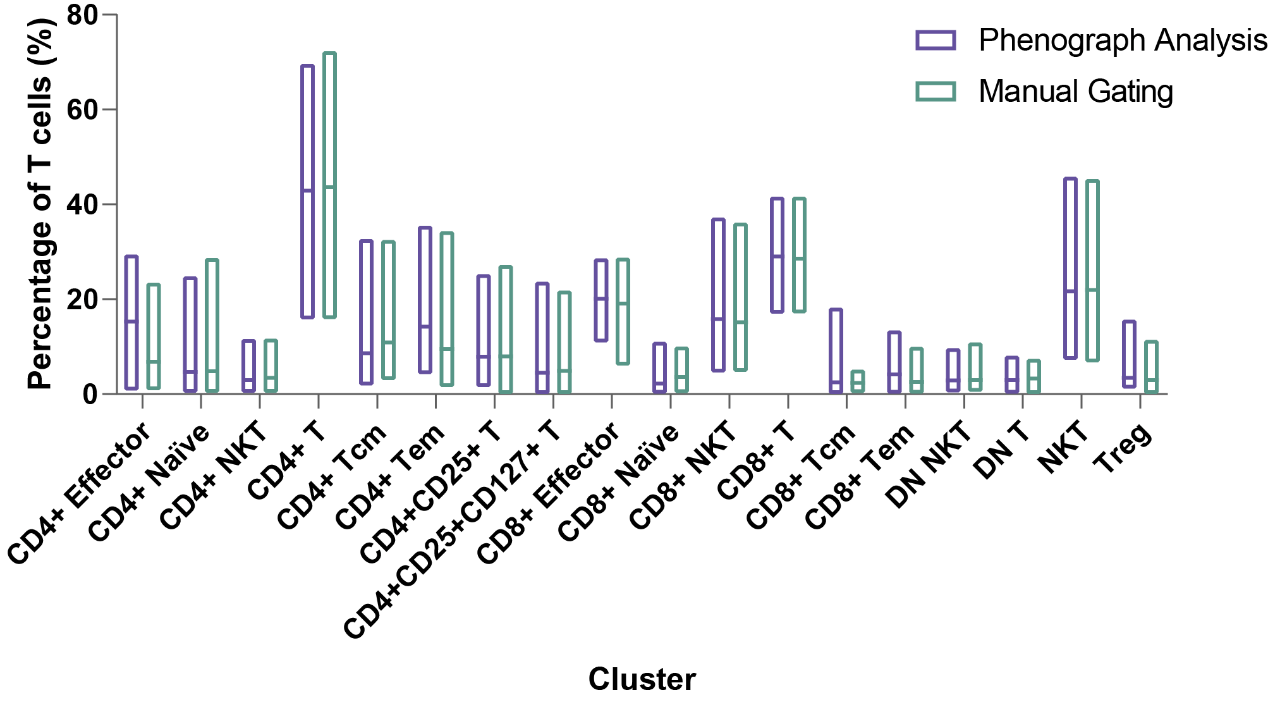


**Figure S8. Frequencies comparison of hand-gated and PhenoGraph-identified cell subsets.** 18 subpopulations were identified by manual gating and PhenoGraph respectively and the bar chart shows frequencies of 18 subpopulations separated by two clustering methods. Group comparisons were performed using unpaired Mann-Whitney test with Benjamini-Hochberg adjustment.


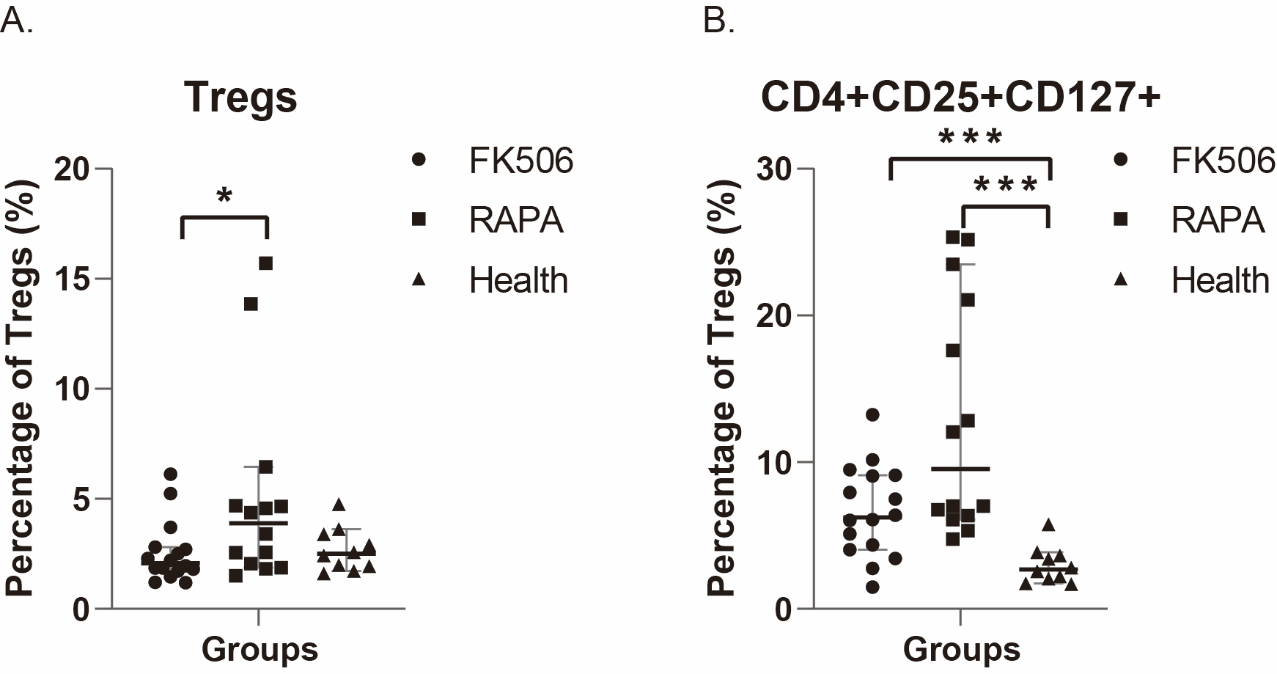


Figure S9. **Frequency comparison of Tregs among the healthy controls, FK506, and RAPA groups.** Comparison of Tregs (cluster 9) and CD4^+^CD25^+^CD127^+^ (merged from clusters 3 and 25) percentages among the healthy controls, FK506, and RAPA groups. Error bars indicate mean ± s.e.m. *q < 0.05, ***q < 0.005. All p-values were calculated using unpaired Mann-Whitney test and q-values were corrected p-values by Benjamini-Hochberg adjustment.


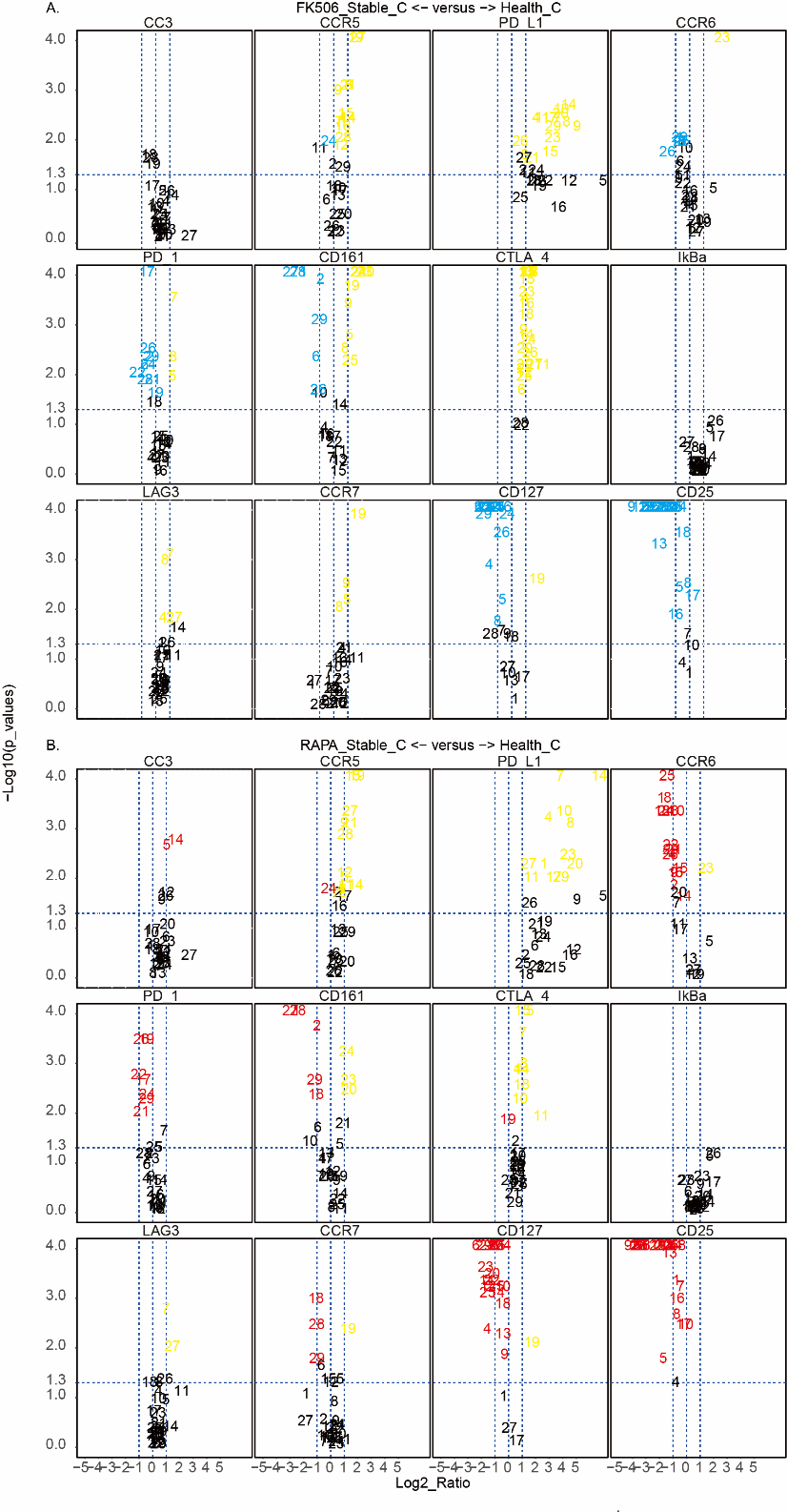


Figure S10. **Volcano plots present distinct functional protein expressions between healthy controls and the FK506 group or the RAPA group.** Volcano plots present significantly differentially expressed (Log_2_ Ratio ≠ 0 and −Log_10_ (p-values) < 1.3 indicates p < 0.05) functional proteins (**A)** between the FK506 group and healthy controls and **(B)** between the RAPA group and healthy controls. The symbols 1-28 represent the 28 clusters identified by PhenoGraph, and 29 represents the entire T-cell lineage. Symbols of significant proteins (q < 0.05) were colored red (RAPA), blue (FK506) or yellow (healthy controls), and non-signiﬁcant functional proteins are colored gray. All p-values were calculated using unpaired Mann-Whitney test and q-values were adjusted p-values with Benjamini-Hochberg correction.


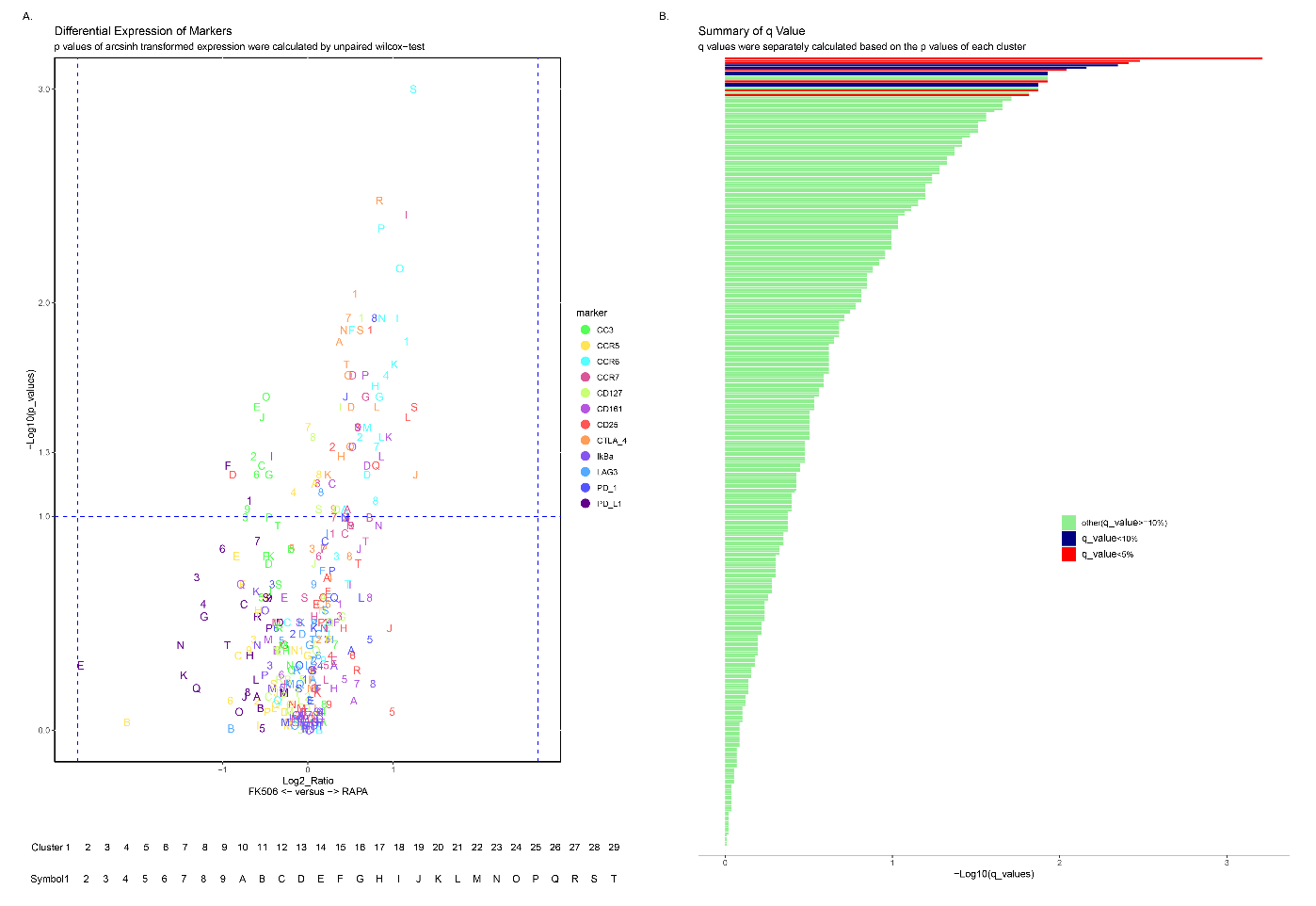


**Figure S11. Volcano plots summary. (A)** The volcano plot showed different protein expression, colored as shown in the legend, between the FK506 and RAPA groups. The symbol represents the cluster number and the letter T represents the entire T-cell lineage. **(B)** Group comparisons between the FK506 and RAPA group were performed using unpaired Mann-Whitney test with Benjamini-Hochberg adjustment.

**
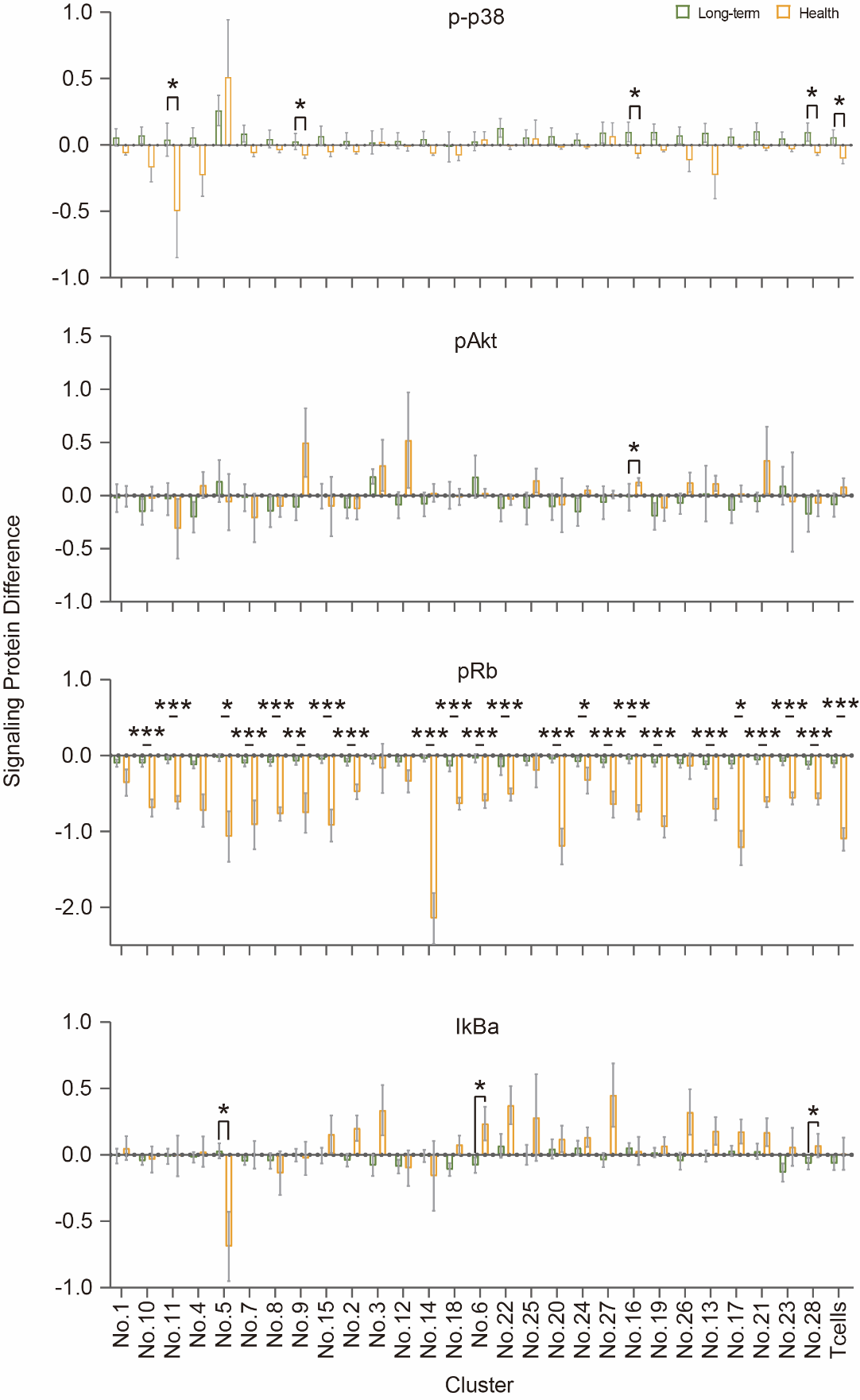
**

**Figure S12. Changes in signaling proteins expression were significantly heterogeneous between the long-term groups and healthy controls.** Stimulus-induced changes in p-p38, pAkt, pRb, and IκBα expression in the 28 clusters identified by PhenoGraph and the entire T-cell lineages were compared between the long-term groups and healthy controls using unpaired Mann-Whitney test with Benjamini-Hochberg adjustment.

**
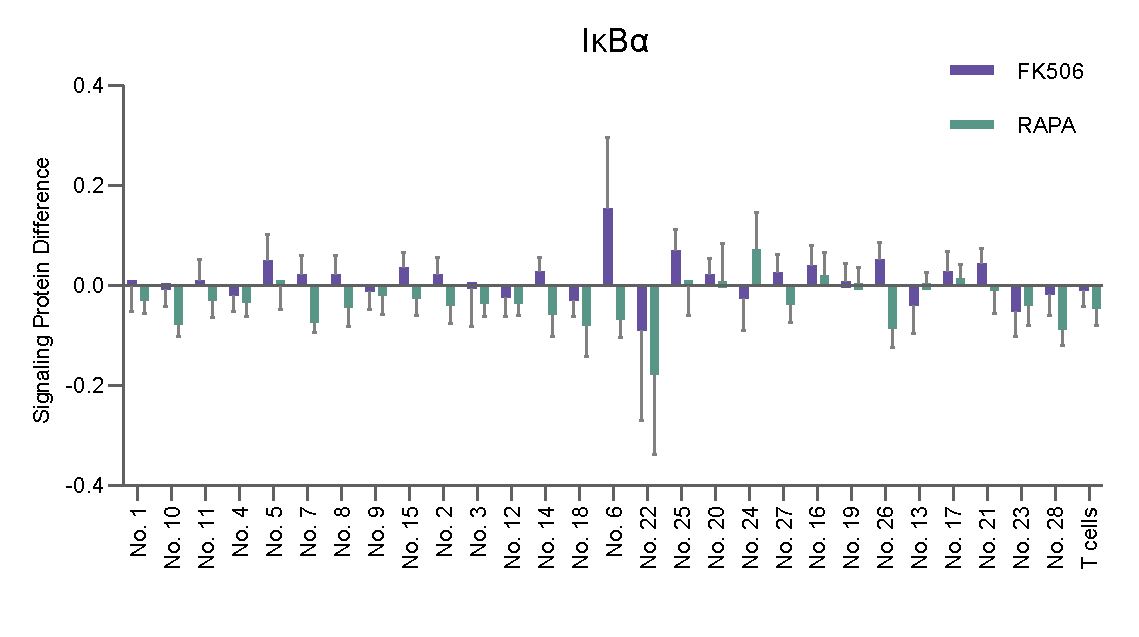
**

**Figure S13. IκBα expression changes were similarly induced by CD3/CD28 between the FK506 and RAPA groups**. Stimulus-induced changes in IκBα expression of 28 clusters identified by PhenoGraph and entire T-cell lineages were compared between the FK506 and RAPA groups using unpaired Mann-Whitney test with Benjamini-Hochberg adjustment.


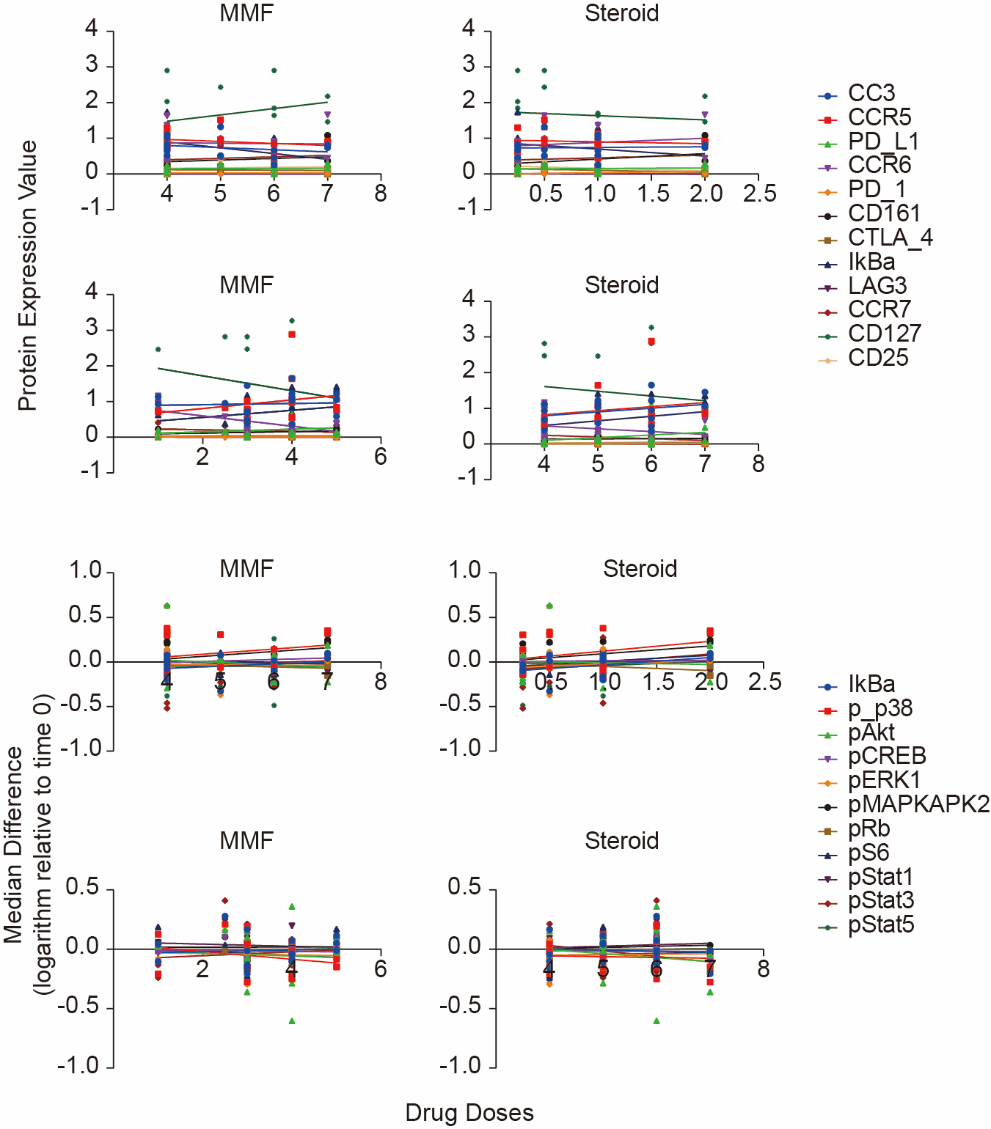


**Figure S14. Pearson correlation between different immunosuppressants and functional protein or signaling responses.** The Pearson correlation method was performed to fit the linear relation between functional protein expression and signaling responses in the T cells and doses of MMF and steroids in the FK506 or RAPA groups.


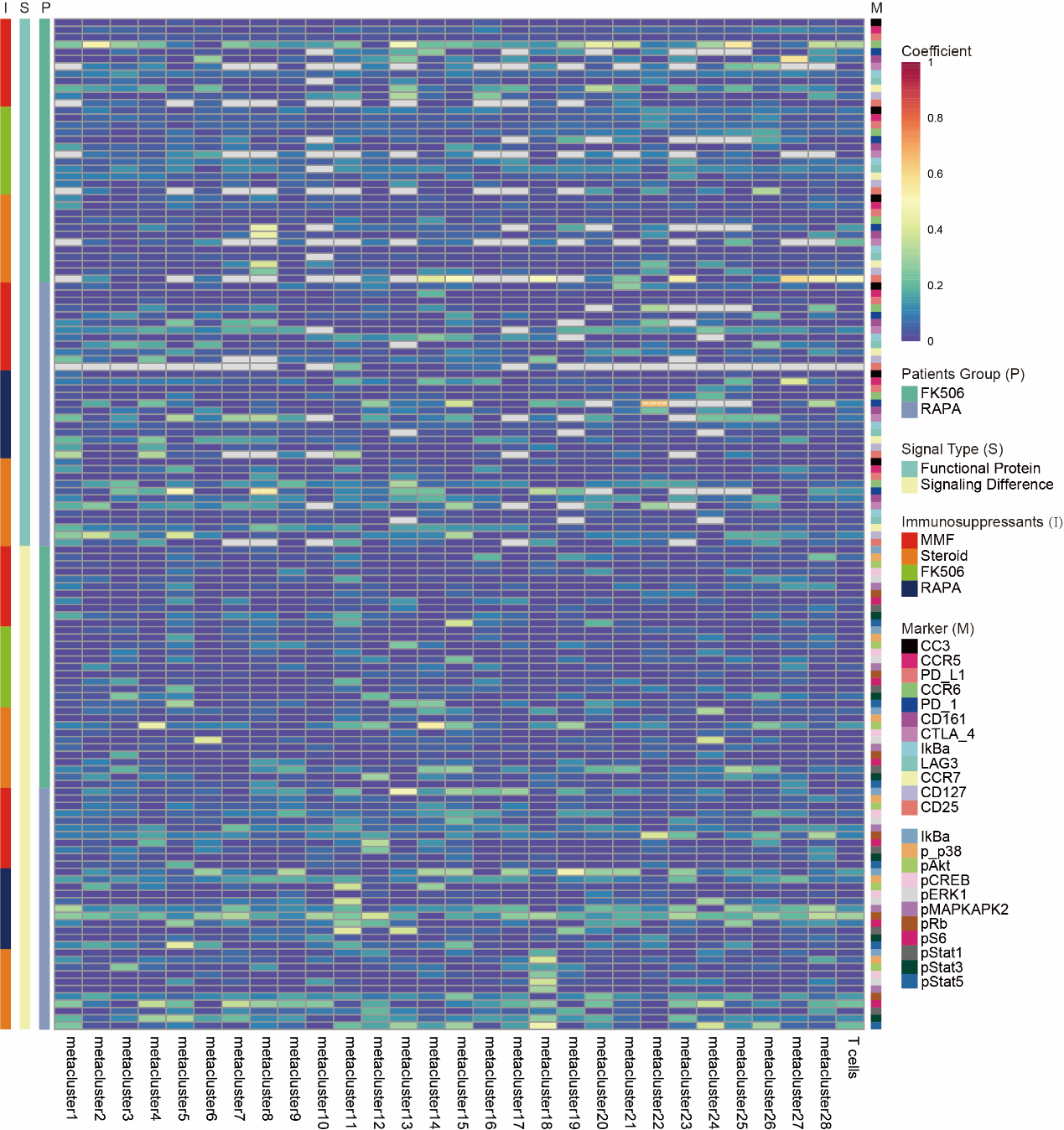


**Figure S15. Pearson correlation coefficients between different immunosuppressants and functional protein or signaling responses.** The Pearson correlation method was performed to fit the linear relation between doses of immunosuppressants and expression of the functional protein or signaling responses and the heatmaps respectively depict the Pearson correlation coefficients in the FK506 and RAPA group.


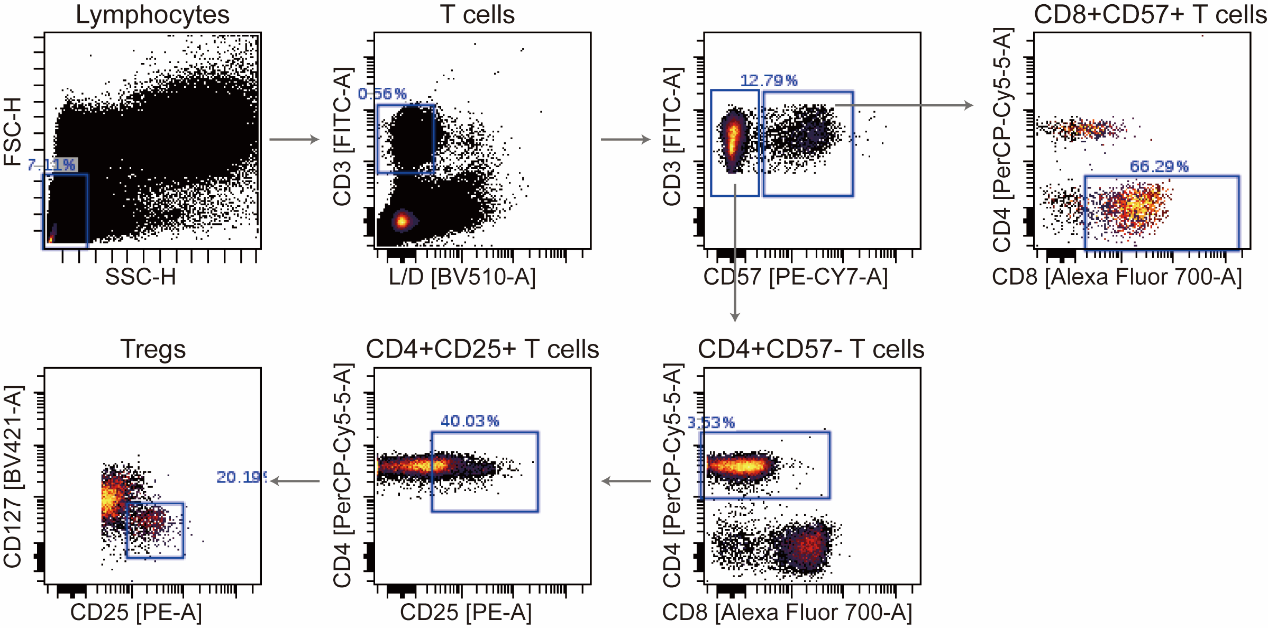


**Figure S16. Manual gating scheme of fluorescent flow cytometry.** A typical gating scheme was used to define Tregs and CD8^+^CD57^+^ T cells. A representative sample was used to display the gating scheme. Tregs: regulatory T cells.
